# An epigenome-wide association study in whole blood of measures of adiposity among Ghanaians: the RODAM study

**DOI:** 10.1186/s13148-017-0403-x

**Published:** 2017-09-21

**Authors:** Karlijn A.C. Meeks, Peter Henneman, Andrea Venema, Tom Burr, Cecilia Galbete, Ina Danquah, Matthias B. Schulze, Frank P. Mockenhaupt, Ellis Owusu-Dabo, Charles N. Rotimi, Juliet Addo, Liam Smeeth, Silver Bahendeka, Joachim Spranger, Marcel M.A.M. Mannens, Mohammad H. Zafarmand, Charles Agyemang, Adebowale Adeyemo

**Affiliations:** 1 Department of Public Health, Academic Medical Center, University of Amsterdam, Meibergdreef 9, 1105AZ Amsterdam, The Netherlands; 2 Department of Clinical Genetics, Academic Medical Center, University of Amsterdam, Meibergdreef 9, 1105AZ Amsterdam, The Netherlands; 3 Source BioScience, 1 Orchard Place, Nottingham Business Park, Nottingham, NG8 6PX UK; 4 Department of Molecular Epidemiology, German Institute of Human Nutrition Potsdam-Rehbruecke, Arthur-Scheunert-Allee 114-116, 14558 Nuthetal, Germany; 5 Institute for Social Medicine, Epidemiology and Health Economics, Charité – Universitaetsmedizin Berlin, Berlin, Germany; 6 Institute of Tropical Medicine and International Health, Charité – University Medicine Berlin, Campus Virchow-Klinikum Augustenburger Platz 1, 13353 Berlin, Germany; 7 Department of Global and International Health, School of Public Health; Kumasi Centre for collaborative Research, College of Health Sciences, Kwame Nkrumah University of Science and Technology, Kumasi, Ashanti Region Ghana; 8 Center for Research on Genomics and Global Health, National Human Genome Research Institute, National Institutes of Health, 12 South Drive, MSC 5635, Bethesda, MD 20892-5635 USA; 9 Department of Non-communicable Disease Epidemiology, London School of Hygiene and Tropical Medicine, Keppel Street, London, WC1E 7HT UK; 10 MKPGMS, Uganda Martyrs University, Kampala, Uganda; 11 Department of Endocrinology and Metabolism, Charité – University Medicine Berlin, Berlin, Germany; 12 German Centre for Cardiovascular Research (DZHK), Berlin, Germany; 13 Center for Cardiovascular Research (CCR), Charité – University Medicine Berlin, Berlin, Germany

**Keywords:** DNA methylation, Africans, Adiposity, Obesity, BMI, Abdominal obesity, Waist circumference, Epigenetic epidemiology, EWAS

## Abstract

**Background:**

Epigenome-wide association studies (EWAS) have identified DNA methylation loci involved in adiposity. However, EWAS on adiposity in sub-Saharan Africans are lacking despite the high burden of adiposity among African populations. We undertook an EWAS for anthropometric indices of adiposity among Ghanaians aiming to identify DNA methylation loci that are significantly associated.

**Methods:**

The Illumina 450k DNA methylation array was used to profile DNA methylation in whole blood samples of 547 Ghanaians from the Research on Obesity and Diabetes among African Migrants (RODAM) study. Differentially methylated positions (DMPs) and differentially methylation regions (DMRs) were identified for BMI and obesity (BMI ≥ 30 kg/m^2^), as well as for waist circumference (WC) and abdominal obesity (WC ≥ 102 cm in men, ≥88 cm in women). All analyses were adjusted for age, sex, blood cell distribution estimates, technical covariates, recruitment site and population stratification. We also did a replication study of previously reported EWAS loci for anthropometric indices in other populations.

**Results:**

We identified 18 DMPs for BMI and 23 for WC. For obesity and abdominal obesity, we identified three and one DMP, respectively. Fourteen DMPs overlapped between BMI and WC. DMP *cg00574958* annotated to gene *CPT1A* was the only DMP associated with all outcomes analysed, attributing to 6.1 and 5.6% of variance in obesity and abdominal obesity, respectively. DMP *cg07839457* (*NLRC5*) and *cg20399616* (*BCAT1*) were significantly associated with BMI, obesity and with WC and had not been reported by previous EWAS on adiposity.

**Conclusions:**

This first EWAS for adiposity in Africans identified three epigenome-wide significant loci (*CPT1A*, *NLRC5* and *BCAT1*) for both general adiposity and abdominal adiposity. The findings are a first step in understanding the role of DNA methylation in adiposity among sub-Saharan Africans. Studies on other sub-Saharan African populations as well as translational studies are needed to determine the role of these DNA methylation variants in the high burden of adiposity among sub-Saharan Africans.

**Electronic supplementary material:**

The online version of this article (10.1186/s13148-017-0403-x) contains supplementary material, which is available to authorized users.

## Background

Adiposity is a major risk factor for non-communicable diseases such as cardiovascular disease and type 2 diabetes [1]. It is more prevalent among African residents in Europe compared with the European host population, in particular among women [2]. Furthermore, both obesity (body mass index (BMI) ≥ 30 kg/m^2^) and abdominal obesity (waist circumference (WC) ≥ 88 cm for women and ≥ 102 cm for men) prevalence have been shown to be much higher in African populations resident in Europe compared with their compatriots in urban and rural Africa. In rural Ghana, the obesity and abdominal obesity prevalence were respectively 5.4 and 19.3% compared with 25.7 and 42.4% in urban Ghana, and 35.6 and 50.5% among Ghanaians resident in Europe [3].

Adiposity results from a wide range of underlying risk factors, including genetic and environmental factors. Genome-wide association studies (GWAS) have identified many genetic risk variants associated with obesity and/or BMI [4]. However, these loci only account for a few percent of variation in BMI [4]. The explained heritability is thought to be low due to a strong effect of environment and lifestyle on BMI [5]. This strong environmental effect is thought to be the “obesogenic” environment, i.e. an environment that predisposes to obesity on a large scale. The obesogenic environment comprises multiple layers such as household, neighbourhood, city and country level [6]. For example, obesogenic attributes on a country level include food availability and exposure to food advertisements. BMI heritability has been shown to be lower in countries with relatively few obesogenic attributes (partly from lower gross domestic product and consumption), as is still the case in many low- and middle-income countries [7].

An important product of gene-environmental interaction is thought to involve epigenetic mechanisms [8]. Epigenetics comprises cellular mechanisms that regulate gene expression, such as chromatin remodelling, histone modifications and DNA methylation [9]. The latter involves the binding of methyl groups to CpG dinucleotides in the DNA and is the most frequent studied epigenetic mechanism. DNA methylation can be influenced by environmental exposures such as climate [10] and by health-related behaviours such as smoking, diet and physical activity [9]. Hence, epigenetics is a means by which genes and environment can interact. Epigenetic modifications, such as DNA methylation changes, can therefore be a consequence of environment, a consequence of genetics or a consequence of both. A defining feature of epigenetics is that it is reversible [10] and could therefore provide potential targets for prevention and intervention. Epigenome-wide association studies (EWAS) have identified epigenetic loci potentially involved in adiposity [11–18]. Despite the increased risk of African ancestry populations for adiposity, only two previous EWAS on adiposity have been conducted in such populations, and these studies were conducted in African Americans [14, 18]. However, African American populations differ from sub-Saharan Africans in environment as well as in genetics (they are an African-European genetically admixed population) [19]. To the best of our knowledge, there are no data on epigenetics in relation to adiposity among sub-Saharan Africans.

In the present study, we aimed to identify epigenetic loci associated with generalized obesity as indicated by BMI and abdominal obesity as indicated by waist circumference (WC), among Ghanaians using the Human Methylation 450K platform of Illumina.

## Methods

### Study population

The present analysis is based on the **R**esearch on **O**besity and **D**iabetes among **A**frican **M**igrants (RODAM) study, of which the study design and data collection are described in detail elsewhere [3, 20]. In brief, the RODAM study collected data between 2012 and 2015 on a relatively homogenous population of sub-Saharan Africans, i.e. Ghanaians. In total, 6385 Ghanaians were recruited from rural Ghana (15 villages in Ashanti region), urban Ghana (Kumasi and Obuasi), London, Amsterdam and Berlin. From the total RODAM study population, a subset of 736 individuals was selected with a BMI range from 15.8 to 51.8 kg/m^2^. For the current analyses, individuals who self-reported having type 2 diabetes (*n* = 128) were excluded. These individuals are more likely to have engaged in weight loss activities because the standard of care and clinical guidelines for type 2 diabetes at the study sites include dietary and exercise intervention both in Ghana [21] and Europe [22]. After additional exclusions (as described below and in the Additional file 1), the final sample size for this analysis was 547. This sample size has over 80% power to detect a 6% difference in methylation with epigenome wide significance between those with generalized obesity (BMI ≥ 30 kg/m^2^) and those without (BMI < 30 kg/m^2^) and an 8% difference in methylation between those with abdominal obesity and those without [23].

### Phenotypic measurements

Information on demographics and self-reported health were collected by self- or interviewer-administered questionnaire. Participants were physically examined, including measurements of weight, height and WC. Height (SECA 217 stadiometer) and weight (SECA 877 scale) were measured in light clothing, and BMI was calculated as weight/height^2^ (kg/m^2^). WC was measured in light clothing at the level midway between the lower rib margin and the iliac crest. Both generalized adiposity and abdominal adiposity were examined. Generalized adiposity was defined by BMI as this is the most wide-spread measure used to assess generalized adiposity. To facilitate interpretation for clinical practice, BMI was dichotomised into a binary generalized adiposity measure—obesity—defined as a BMI of ≥ 30.0 kg/m^2^ according to the World Health Organization (WHO) definition [24]. WC was used as measure for abdominal obesity and was dichotomised following WHO recommendations, i.e. abdominal obesity is WC ≥ 88 cm for women and ≥ 102 cm for men [25].

### DNA profiling, processing and quality control

Fasting blood samples were collected by trained research assistants and shipped to Source Bioscience Nottingham for DNA extraction, genotyping and DNA methylation profiling.

Bisulfite treatment of DNA (Zymo EZ DNA MethylationTM kit) was used to deaminate unmethylated cytosine to produce uracil in DNA conform manufacturer’s protocol. The converted DNA was amplified and hybridized on the Illumina Human Methylation 450K array which quantifies DNA methylation levels of approximately 485,000 CpG sites. The samples were divided over eight bisulfite conversion and hybridization batches. Raw 450K data were processed for primary quality control using the statistical software platform “R” (version 3.2.2) and the *MethylAid* package (version 1.4.0). An overview of R packages used can be found in Additional file 1: Table S1. *MethylAid* detects poor-quality samples using sample-dependent and sample-independent control CpG sites present on the 450K array itself [26]. MethylAid threshold values included methylated and unmethylated intensities of 10.5, overall quality control of 11.75, bisulfite control of 12.75, hybridization control of 12.50 and a detection *p* value of 0.95. Based on these thresholds, 12 samples were considered outliers (Additional file 1: Figure S1-B).

To check for potential population stratification, principal component analysis (PCA) was done using PLINK 1.9 [27] on genotypes obtained from the African Diaspora Power Chip (ADPC). Evaluation of the scree plot (Additional file 1: Figure S1-A) combined with formal testing for significant PCs using the *minimum average partial test* [28] revealed only PC 1 as a significant PC. This first PC was included in the genome-wide epigenetic association models to adjust for possible residual population stratification. Although PC 2 and PC 3 accounted for moderate amounts of the total variance, the addition of PC 2 and PC 3 in association models did not further improve or significantly alter our results. Therefore, PC 2 and PC 3 were not included in our final models (data not shown). Genotyping data (not reported here) revealed eight samples with a sex discordance compared with the phenotype data that were subsequently excluded.

Functional normalization was applied using the “R” *minfi* package to normalize raw 450K data. PCA on the normalized 450K dataset annotated for sex, recruitment site, self-reported ethnic group within Ghana, bisulfite batch, hybridization batch and array position revealed three additional gender-discordant samples and some stratification on array position. No other outliers were observed in the epigenetics PCA. Sex-discordant samples detected by genetic and/or epigenetic analyses were removed. All nonspecific CpG sites were removed [29] as well as CpG sites located on chromosomes X and Y. Removal of these CpG sites resulted in a set of 429,459 CpG sites which were used to identify differentially methylated positions (DMPs) and differentially methylated regions (DMRs) in linear regression analysis as described below.

Cell composition of whole blood samples is a source of variability in DNA methylation and has thereby the potential to cause confounding [30]. We therefore estimated cell distributions using the method proposed by Houseman et al. [31] with the reference population as proposed by Reinius et al. [32] and included estimated cell type distributions as covariate in the analyses. Additional file 1: Figure S1-C shows the correlation between the blood cell distribution estimates and PC 1 to 8 of the EWAS. Although observed correlations between cell type estimates and any PC (Additional file 1: Figure S1-C) were weak, cell distributions were added to the models as covariate because cell distribution bias remains likely to be present according to previous reports [31, 33]. The weak correlation between cell type estimates and the PCs is likely to be due to presence of other, stronger, confounding factors affecting both the CpGs involved in cell non-mediated and mediated processes. Additional file 1: Figure S1-D shows the correlation between the other covariates and principal component 1 to 8 of the PCA performed on the normalized 450K data. Since previous reports have shown a potential link between blood cell distribution and adiposity parameters [34], we performed multicollinearity analyses. These analyses showed a tolerance statistic and a variance inflation factor (VIF) of both 1.0. We have therefore no indication for multicollinearity between cell distribution estimates and adiposity indices.

### Statistical analysis

#### Differentially methylated positions

Linear regression analyses were performed in “R” with the *minfi* package using DNA methylation levels as dependent variable to identify DMPs for BMI and WC, as well as for obesity and abdominal obesity. Age, sex, recruitment site, estimated cell distributions, technical effects (hybridization batch and array position) and the first principal component from genotyping data were included as covariates. Model fitting was evaluated using QQ-plots (Additional file 1: Figure S2). In GWAS, results are generally corrected for (genomic) inflation. In order to address inflation of our analyses, we applied the recently reported EWAS method by Iterson et al. [35] (Additional file 1: File S3). False discovery rate (FDR) adjustment was used to adjust for multiple testing. A FDR of 0.05 was considered epigenome-wide significant. DMPs identified in relation to obesity and abdominal obesity were subsequently included in logistic regression models to assess the odds for obesity and abdominal obesity per 1% increase in DNA methylation of respective DMPs. The Nagelkerke’s R-squared statistic from the logistic regression models with and without covariates was used to calculate trait variance explained by a locus. The region around these DMPs was visualized using the *coMET “*R” package [36]*.*

For all DMP analyses, *M* values were calculated as the log2 ratio of the intensities of methylated CpG site versus unmethylated CpG site. Significant differences were determined based on *M* values instead of beta values as is recommended by Du et al. [37]. Corresponding beta values were reported to facilitate biological interpretation [37].

#### Differentially methylated regions

To find DMRs, we fitted similar models to DMP analyses using the *bumphunter* function in the *minfi* package [38]. We calculated the DMRs with the methylation cut-off of 0.015 (corresponding to 1.5% difference in beta values) for obesity and abdominal obesity and with cut-off 0.0015 and 0.0005 for BMI and WC, respectively. The DMR methylation cut-offs were optimized based on observed effect sizes and significance levels in a volcano plot. Estimates were done using bootstrap with 500 permutations. We defined a DMR as three or more CpG sites within one cluster. Multiple testing adjustment was performed using family-wise error rate (FWER). We filtered CpG sites with FWER < 0.2.

#### Replication of loci reported in non-African populations

Previously reported loci for multiple anthropometric indices for adiposity were identified through a literature search. PUBMED was systematically searched in April 2016 for all papers on DNA methylation and adiposity published between January 2013 and April 2016. The search included keywords and MeSH terms on obesity, BMI, abdominal obesity, WC, DNA methylation and for exclusion keywords on animal models (Additional file 1: File S4). The search resulted in 498 hits (Additional file 1: Figure S4). Titles and abstracts were considered eligible when written in English, the reported outcome was related to obesity, sample size was at least 10 and data were derived from humans or human cell lines. Title and abstract screening was performed independently by two co-authors (PH and KM). All conflicts were discussed and resolved before proceeding to the next stage. Abstract screening resulted in 52 articles for full-text screening and data extraction. Twenty-six papers were excluded because their full texts did not report self-generated results, found no significant associations or the studies were not directly related to adiposity outcomes. For each study reporting candidate CpG sites (*n* = 26), the following data were extracted: authors, year of publication, ethnicity, sample size, tissue, candidate gene, analysis technique, chromosome, position, candidate CpG site, hypo- or hyper-methylation and trait. After removal of duplicates, 1027 unique 450k array CpG sites remained (Additional file 1: File S5).

#### Pathway enrichment analysis

Pathway analyses were performed using the “WEB-based GEneSeT AnaLysis Toolkit” (WebGestalt) (www.webgestalt.org). We used the tool to evaluate Biological Process, Cellular Component and Molecular Function pathways from the GO terms for the top 100 of the BMI and WC analyses separately. The following settings were applied: GO database, hsapiens_gene_symbol, hypergeometric statistical method, FDR multiple test adjustment, significance level *q* < 0.05, minimum number of genes for category = 4.

## Results

### Participant characteristics

The 547 individuals in the analyses had a mean BMI of 26.7 kg/m^2^ (95% CI 26.2–27.2) and a mean WC of 90.3 cm (95% CI 89.3–91.4) (Table 1). The analysis included 290 individuals with obesity and 257 without obesity. By WC criteria, 422 participants had abdominal obesity and 164 had normal WC. Those with and without obesity were similar in age and had a similar distribution of men and women. Those with abdominal obesity were similar in age as compared with those without but were more often female. Blood cell distribution estimates did not differ between those with and without obesity and abdominal obesity.

**Table 1:** Characteristics of participants included in the analyses

	All	Obesity	No obesity	Abdominal obesity	No abdominal obesity
*n*	(*n* = 547)	(*n* = 290)	(*n* = 257)	(*n* = 422)	(*n* = 164)
Age, years	50.5 (49.7–51.3)	50.7 (49.6–51.8)	50.4 (49.2–51.6)	50.4 (49.0–51.8)	50.6 (49.6–51.6)
Male, %	42.2 (38.1–46.4)	50.2 (43.8–56.7)	49.8 (43.3–56.2)	15.2 (11.1–20.4)	84.8 (79.6–88.9)
BMI, kg/m^2^	26.7 (26.2–27.2)	30.9 (30.5–31.4)	21.9 (21.7–22.2)	31.9 (31.3–32.5)	23.6 (23.3–24.0)
Waist circumference, cm	90.3 (89.3–91.4)	99.4 (98.4–100.5)	80.1 (79.3–80.8)	102.1 (100.9–103.3)	83.4 (82.5–84.3)
Site, %					
Rural Ghana	13.9 (11.2–17.1)	5.5 (3.4–8.8)	23.3 (18.6–28.9)	8.9 (5.6–13.7)	16.9 (13.3–21.2)
Urban Ghana	36.4 (32.4–40.5)	31.7 (26.6–37.3)	41.6 (35.7–47.8)	38.4 (32.0–45.3)	35.2 (30.3–40.4)
Amsterdam	20.3 (17.1–23.9)	24.1 (19.5–29.4)	9.7 (6.6–14.0)	21.2 (16.1–27.4)	19.8 (15.9–24.3)
Berlin	10.2 (8.0–13.1)	11.0 (7.9–15.2)	9.3 (6.3–13.6)	5.9 (3.4–10.1)	12.8 (9.6–16.8)
London	19.1 (16.1–22.7)	27.6 (22.7–33.0)	16.0 (12.0–21.0)	25.6 (20.1–32.1)	15.4 (12.0–19.6)
Cell counts, %					
CD8+ T cells	10.9 (10.5–11.3)	10.8 (10.3–11.4)	11.1 (10.4–11.7)	10.8 (10.1–11.4)	11.0 (10.5–11.6)
CD4+ T cells	18.5 (18.0–19.0)	18.3 (17.7–18.9)	18.7 (18.0–19.4)	18.2 (17.5–19.0)	18.6 (18.0–19.2)
Natural killer cells	10.9 (10.5–11.4)	11.1 (10.4–11.8)	10.7 (10.1–11.4)	10.8 (10.0–11.6)	11.0 (10.4–11.6)
B cells	10.7 (10.5–11.0)	10.7 (10.3–11.1)	10.8 (10.4–11.2)	10.7 (10.2–11.2)	10.8 (10.4–11.1)
Monocytes	7.9 (7.7–8.1)	7.9 (7.6–8.2)	7.9 (7.6–8.2)	7.9 (7.6–8.3)	7.9 (7.6–8.1)
Granulocytes	44.8 (44.0–45.5)	45.0 (43.9–46.1)	44.4 (43.3–45.5)	45.5 (44.1–46.7)	44.4 (43.4–45.3)

Numbers are in means or percentages with corresponding (confidence intervals)

### Differentially methylated positions

**Fig. 1:**
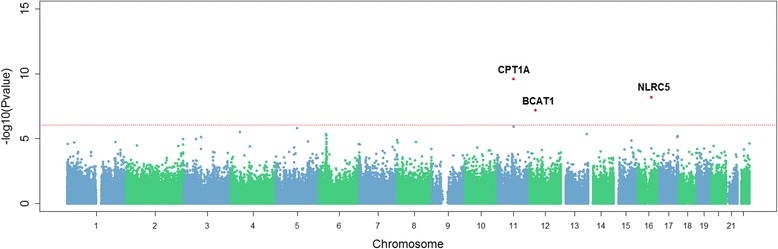
Manhattan plot of epigenome wide *p* values for obesity (BMI ≥ 30 kg/m^2^). The red dotted line indicates epigenome-wide significance according to FDR multiple test correction

Eighteen DMPs were found associated with BMI as a continuous variable (Table 2 and Additional file 1: Figure S6). In contrast, only three DMPs—*cg00574958, cg07839457* and *cg20399616—*, annotated to genes *CPT1A, NLRC5* and *BCAT1*, respectively*,* were significantly differentially methylated in those with obesity compared with those without (Table 2 and Fig. 1). The majority of DMPs (15 out of 18) were hyper-methylated for higher BMIs. Hypo-methylated DMPs for higher BMI were annotated to genes *CPT1A, BCAT1* and *HTRA1*. The DMPs annotated to *CPT1A* and *BCAT1* were also significantly hypo-methylated in those with obesity compared with those without.

**Table 2:** Differentially methylated positions for BMI and obesity (BMI ≥ 30 kg/m^2^)

					BMI			Obesity		
CpG site	CHR	Position	Nearest gene^a^	Feature^b^	Delta *β* value^c^	*p* value^d^	FDR^d^	Delta *β* value^c^	*p* value^d^	FDR^d^
cg07839457	16	57023022	*NLRC5*	TSS1500	0.0071	3.79E − 11	1.63E − 05	0.0711	6.38E − 09	1.37E − 03
cg08818207	6	32820355	*TAP1*	Body	0.0047	7.66E − 08	1.64E − 02	0.0440	5.61E − 06	2.67E − 01
cg00574958	11	68607622	*CPT1A*	5′ UTR	− 0.0014	1.45E − 07	1.71E − 02	− 0.0189	2.53E − 10	1.09E − 04
cg08099136	6	32811251	*PSMB8*	Body	0.0033	1.59E − 07	1.71E − 02	0.0321	1.18E − 04	6.62E − 01
cg01309328	6	32811253	*PSMB8*	Body	0.0029	3.38E − 07	2.91E − 02	0.0315	1.02E − 05	3.19E − 01
cg20399616	12	25055967	*BCAT1*	Body	− 0.0028	4.61E − 07	3.30E − 02	− 0.0297	6.36E − 08	9.10E − 03
cg22107533	15	45028083	*TRIM69*	TSS1500	0.0037	5.69E − 07	3.49E − 02	0.0330	7.10E − 05	6.41E − 01
cg06820412	5	135386296	*TGFBI*	Body	0.0005	7.22E − 07	3.81E − 02	0.0050	1.63E − 05	3.71E − 01
cg00218406	6	31431407	*HCP5*	3′ UTR	0.0051	8.03E − 07	3.81E − 02	0.0483	4.34E − 05	5.33E − 01
cg25954539	6	31323677	*HLA-B*	Body	0.0046	1.02E − 06	3.81E − 02	0.0454	2.70E − 05	4.15E − 01
cg23235965	6	30459540	*HLA-E*	Body	0.0026	1.04E − 06	3.81E − 02	0.0270	4.50E − 06	2.42E − 01
cg25178683	17	76976267	*LGALS3BP*	TSS1500	0.0019	1.08E − 06	3.81E − 02	0.0195	7.91E − 05	6.41E − 01
cg08996521	3	50649994	*CISH*	TSS1500	0.0015	1.26E − 06	3.81E − 02	0.0142	1.06E − 05	3.19E − 01
cg04927537	17	76976091	*LGALS3BP*	TSS200	0.0026	1.34E − 06	3.81E − 02	0.0286	6.46E − 06	2.76E − 01
cg05490029	8	79719015	*IL7*	TSS1500	0.0026	1.45E − 06	3.81E − 02	0.0244	1.80E − 05	3.71E − 01
cg18954700	10	124220854	*HTRA1*	TSS200	− 0.0015	1.49E − 06	3.81E − 02	− 0.0137	4.74E − 04	8.19E − 01
cg25843003	6	31431312	*HCP5*	3′ UTR	0.0021	1.51E − 06	3.81E − 02	0.0200	5.98E − 05	6.22E − 01
cg06118217	2	240100998	*HDAC4*	Body	0.0008	1.72E − 06	4.11E − 02	0.0069	1.11E − 05	3.19E − 01

^a^CpGs are located in the gene if no distance is indicated

^b^Based on manifest feature annotation Illumina. IGR, intergenic region

^c^Negative beta values indicate lower DNA methylation (hypo-methylation) in cases compared with controls

^d^ *p* values and FDR corresponding to *M* values. Table is sorted on BMI associated *p* values. All significant hits for both outcomes are included

**Fig. 2:**
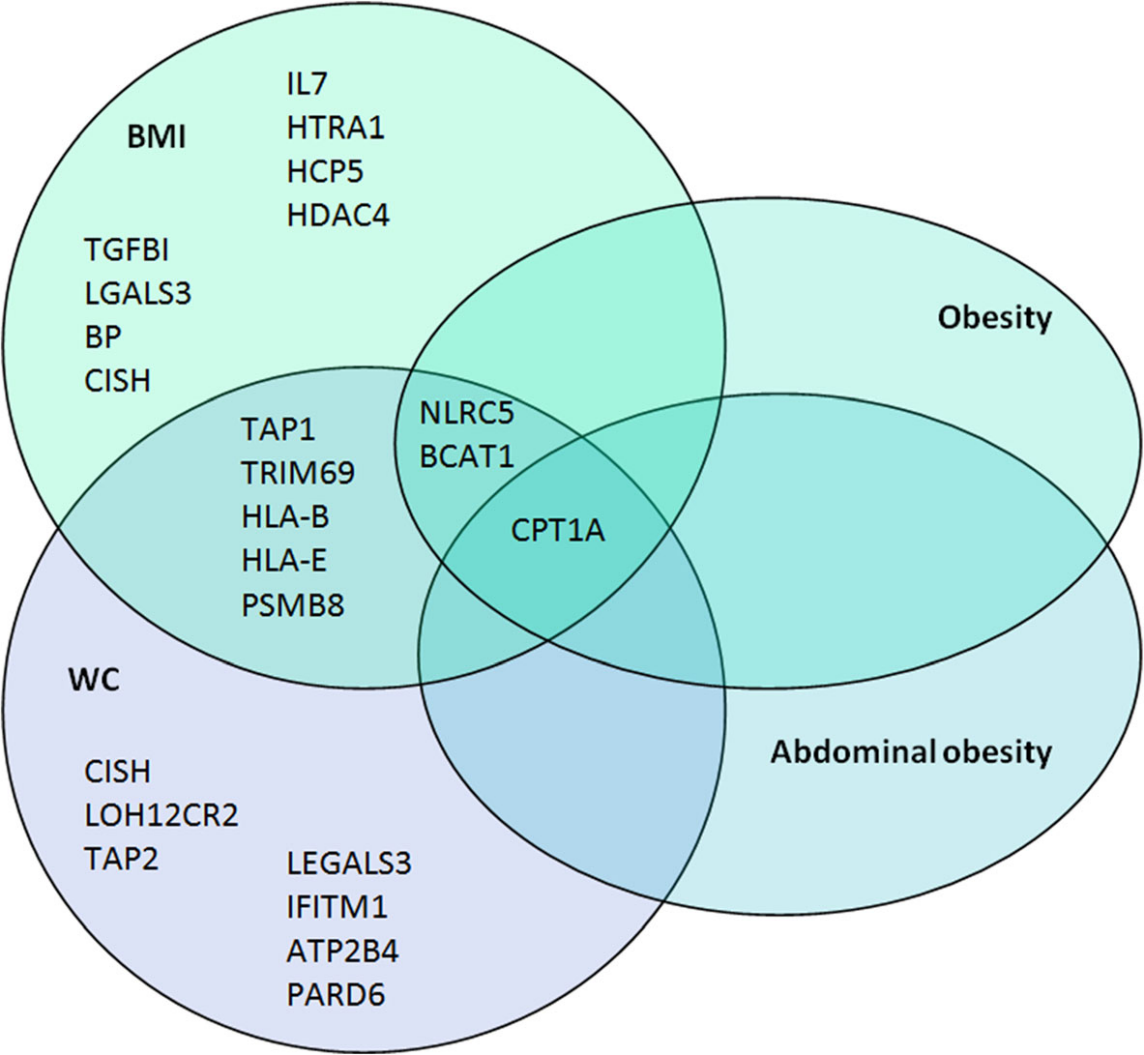
Venn diagram of overlapping genes in or near differentially methylated positions (DMPs) in relation to BMI, WC, obesity and abdominal obesity

**Fig. 3:**
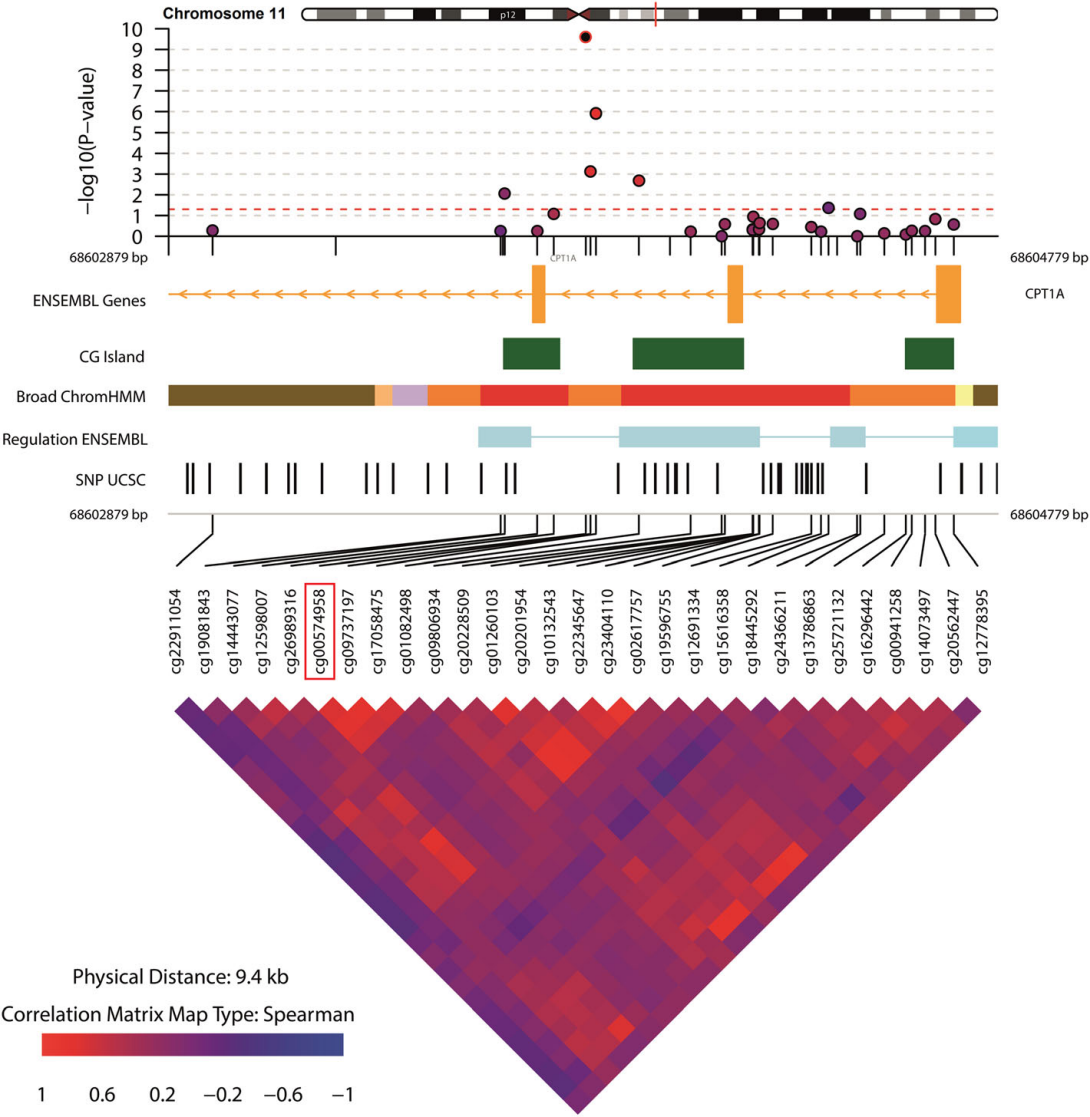
Differentially methylated position (DMP) annotated to gene *CPT1A* associated with BMI, WC, obesity and abdominal obesity. The DMP is annotated as the CpG site in the red square. A 5-kb region around the DMP was visualized

**Fig. 4:**
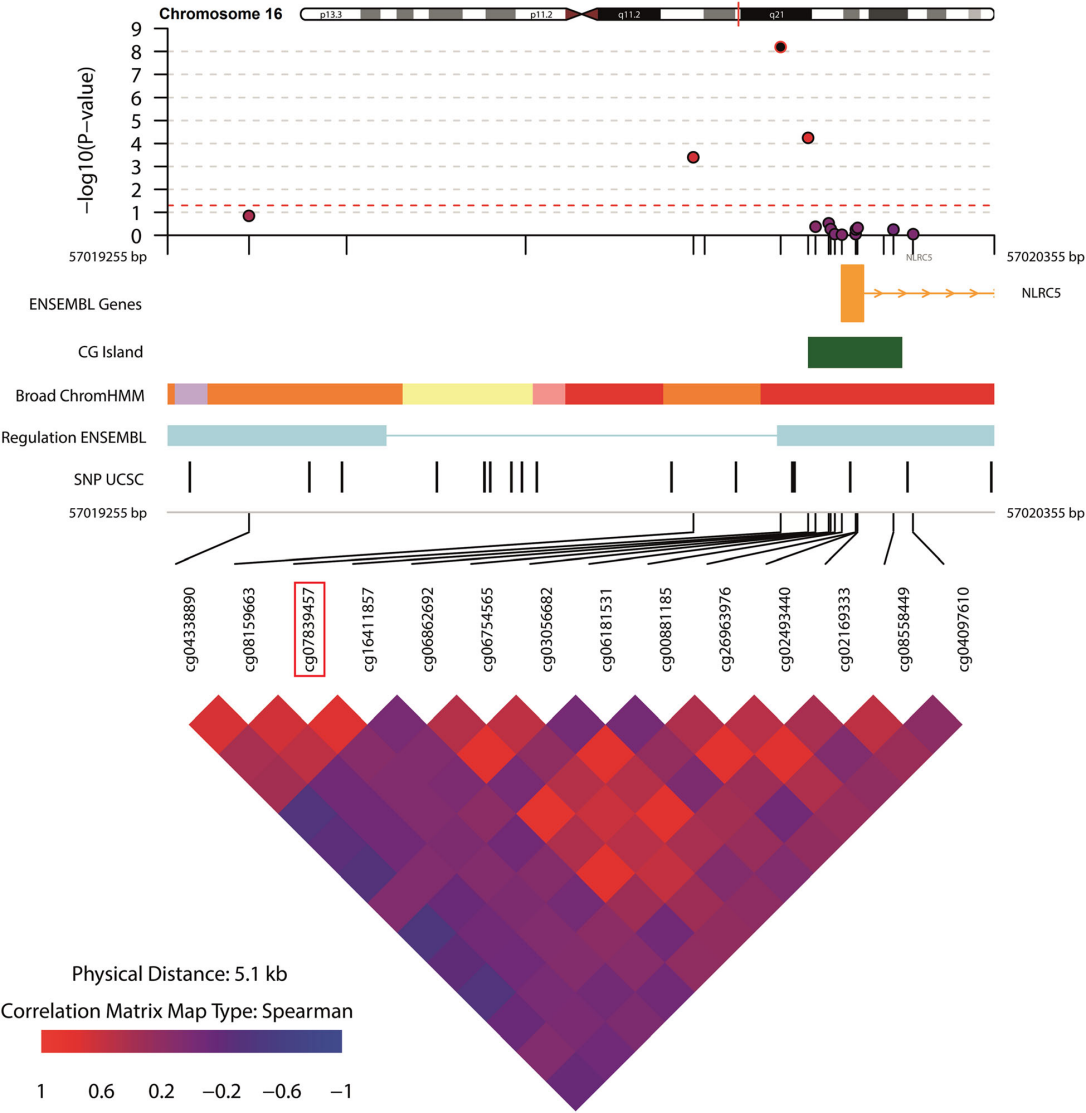
Differentially methylated position (DMP) annotated to gene *NLRC5* associated with BMI, obesity and WC. The DMP is annotated as the CpG site in the red square. A 5-kb region around the DMP was visualized

**Fig. 5:**
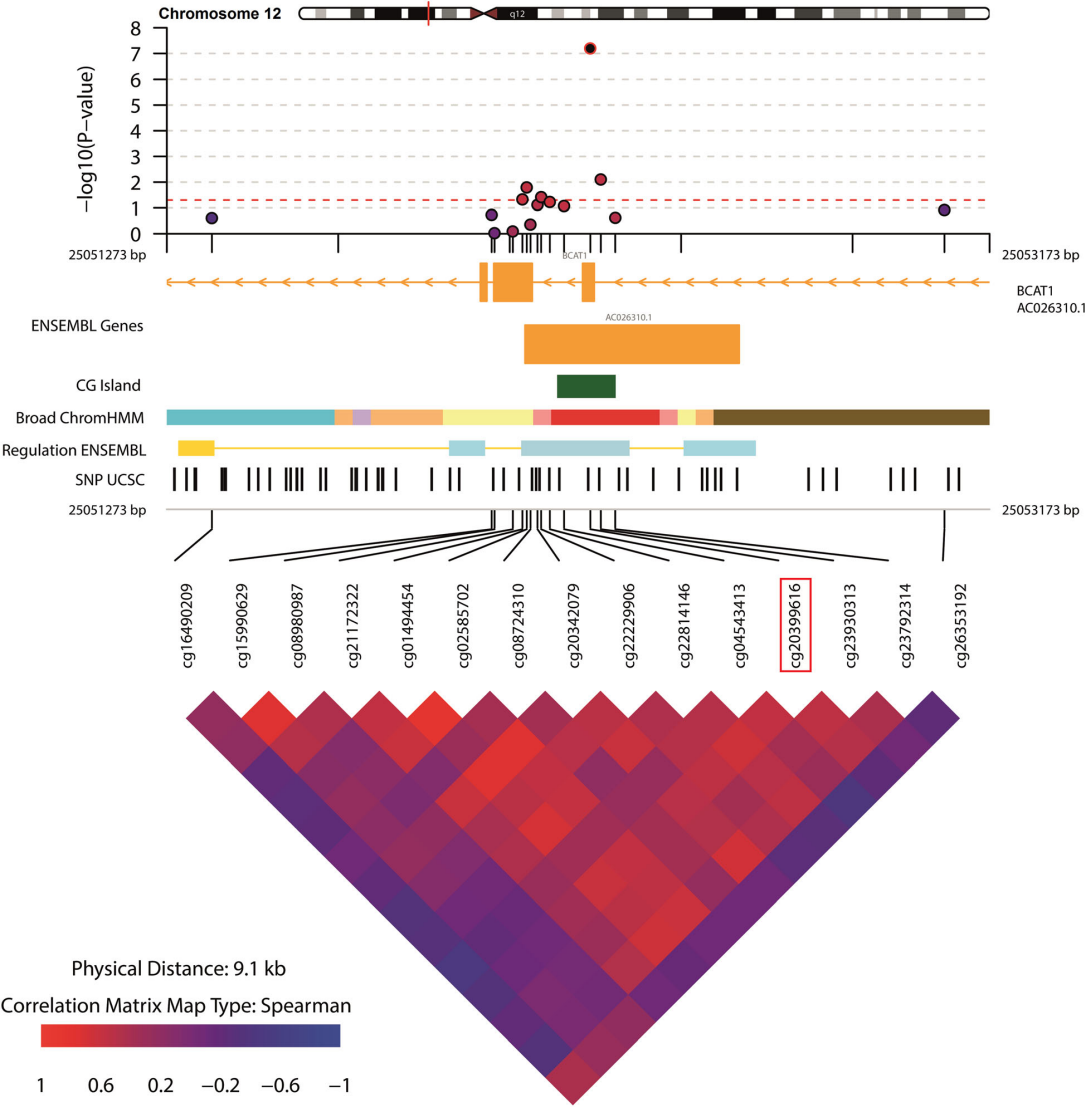
Differentially methylated position (DMP) annotated to gene *BCAT1* associated with BMI, obesity and WC. The DMP is annotated as the CpG site in the red square. A 5-kb region around the DMP was visualized

Twenty-three DMPs were associated with WC of which the majority was hyper-methylated in relation to a higher WC (Table 3 and Additional file 1: Figure S7). Figure 2 shows the overlap in DMPs for BMI, WC, obesity and abdominal obesity. The direction of effect for these DMPs was consistent between adiposity indices. The odds for the obesity were 0.85, 1.04 and 0.94 for the DMPs annotated to genes *CPT1A*, *NLRC5* and *BCAT1*, respectively (Table 4). The DMP annotated to gene *CPT1A*, which was significantly hypo-methylated in individuals with obesity, was also significantly hypo-methylated in individuals with abdominal obesity compared with those without (Table 3 and Additional file 1: Figure S8), with similar odds (OR = 0.84, 95% CI 0.79–0.90) for abdominal obesity as for obesity (Table 4). Implementation of the *BACON* method for reducing inflation (Additional file 1: File S3) did in general not result in a decrease of observed inflation (Additional file 1: Figure S3). In addition, the inflation-uncorrected and inflation-corrected FDR adjusted *p* values did not differ substantially, i.e. the observed genome-wide significant DMPs remained the same (Additional file 1: Table S3-A and Table S3-B). The DMPs annotated to genes *CPT1A*, *NLRC5* and *BCAT*1, which were associated with three out of four anthropometric indices studied, combined attributed to 7.6% of the variance on obesity. 6.1% of variation in obesity was attributable to *CPT1A* alone and 5.6% for abdominal obesity (Table 4). Figures 3, 4 and 5 visualize the region around the DMPs annotated to *CPT1A*, *NLRC5* and *BCAT1*. Adjustment for energy intake and physical activity did not substantially alter the results (Additional file 1: File S9).

**Table 3:** Differentially methylated positions for WC and abdominal obesity (WC ≥ 88 cm for women and ≥ 102 cm for men)

					WC			Abdominal obesity		
CpG site	CHR	Position	Nearest gene^a^	Feature^b^	Delta *β* value^c^	*p* value^d^	FDR^d^	Delta *β* value^c^	*p* value^d^	FDR^d^
cg07839457	16	57023022	*NLRC5*	TSS1500	0.0030	2.24E − 10	9.61E − 05	0.0340	6.86E − 05	8.17E − 01
cg00574958	11	68607622	*CPT1A*	5′ UTR	− 0.0007	1.00E − 08	1.55E − 03	− 0.0135	1.52E − 08	6.54E − 03
cg25954539	6	31323677	*HLA-B*	Body	0.0022	1.08E − 08	1.55E − 03	0.0316	5.78E − 05	7.75E − 01
cg08818207	6	32820355	*TAP1*	Body	0.0020	3.46E − 08	3.71E − 03	0.0303	5.14E − 05	7.75E − 01
cg04927537	17	76976091	*LGALS3BP*	TSS200	0.0013	6.19E − 08	5.08E − 03	0.0195	4.23E − 05	7.75E − 01
cg22107533	15	45028083	*TRIM69*	TSS1500	0.0015	7.76E − 08	5.08E − 03	0.0252	2.14E − 04	8.17E − 01
cg01309328	6	32811253	*PSMB8*	Body	0.0014	8.69E − 08	5.08E − 03	0.0202	6.26E − 06	6.45E − 01
cg23533285	6	31322348	*HLA-B*	Body	0.0015	9.47E − 08	5.08E − 03	0.0241	2.99E − 04	8.17E − 01
cg23570810	11	315102	*IFITM1*	Body	0.0018	1.12E − 07	5.35E − 03	0.0182	1.12E − 05	6.68E − 01
cg00218406	6	31431407	*HCP5*	3′ UTR	0.0022	1.66E − 07	7.12E − 03	0.0335	7.64E − 04	8.17E − 01
cg08099136	6	32811251	*PSMB8*	Body	0.0015	1.93E − 07	7.53E − 03	0.0180	3.23E − 04	8.17E − 01
cg11202345	17	76976057	*LGALS3BP*	1stExon	0.0011	2.15E − 07	7.70E − 03	0.0203	4.65E − 05	7.75E − 01
cg25178683	17	76976267	*LGALS3BP*	TSS1500	0.0011	3.50E − 07	1.16E − 02	0.0136	1.41E − 05	6.68E − 01
cg01971407	11	313624	*IFITM1*	TSS1500	0.0010	4.69E − 07	1.44E − 02	0.0079	5.71E − 05	7.75E − 01
cg25843003	6	31431312	*HCP5*	3′ UTR	0.0009	7.03E − 07	2.01E − 02	0.0142	8.12E − 05	8.17E − 01
cg22940798	6	32805554	*TAP2*	Body	0.0010	1.03E − 06	2.75E − 02	0.0187	5.19E − 04	8.17E − 01
cg20399616	12	25055967	*BCAT1*	Body	− 0.0012	1.20E − 06	2.97E − 02	− 0.0145	9.27E − 04	8.17E − 01
cg05439368	15	45028098	*TRIM69*	TSS1500	0.0015	1.25E − 06	2.97E − 02	0.0263	1.99E − 04	8.17E − 01
cg06538684	12	12511223	*LOH12CR2*	TSS1500	0.0011	1.62E − 06	3.53E − 02	0.0173	9.31E − 05	8.17E − 01
cg23235965	6	30459540	*HLA-E*	Body	0.0011	1.64E − 06	3.53E − 02	0.0238	5.37E − 05	7.75E − 01
cg13558971	1	203597085	*ATP2B4*	5′ UTR	− 0.0007	1.78E − 06	3.64E − 02	− 0.0092	1.82E − 03	8.17E − 01
cg13348877	18	78005237	*PARD6G*	1stExon	− 0.0002	2.01E − 06	3.93E − 02	− 0.0021	8.15E − 05	8.17E − 01
cg08996521	3	50649994	*CISH*	TSS1500	0.0005	2.52E − 06	4.70E − 02	0.0131	1.10E − 04	8.17E − 01

^a^CpGs are located in the gene if no distance is indicated

^b^Based on manifest feature annotation Illumina. IGR, intergenic region

^c^Negative beta values indicate lower DNA methylation (hypo-methylation) in cases compared with controls

^d^ *p* values and FDR corresponding to *M* values. Table is sorted on WC associated *p* values. All significant hits for both outcomes are included

**Table 4:** Odds ratios for obesity and abdominal obesity Differentially Methylated Positions (DMPs)

					Obesity			Abdominal obesity		
CpG	CHR	Position	Gene^a^	Feature^b^	OR^c^	95% CI	Attributable trait variance (%)	OR^c^	95% CI	Attributable trait variance (%)
cg07839457	16	57023022	*NLRC5*	TSS1500	1.04	1.02–1.06	2.4	1.03	1.01–1.06	1.4
cg00574958	11	68607622	*CPT1A*	5′ UTR	0.85	0.80–0.91	6.1	0.84	0.79–0.90	5.6
cg20399616	12	25055967	*BCAT1*	Body	0.94	0.90–0.98	1.6	0.96	0.91–1.00	0.7

^a^CpGs are located in the gene if no distance is indicated

^b^Based on manifest feature annotation Illumina

^c^Odds ratios are per 1% increase in DNA methylation

The DMPs *cg07839457* (*NLRC5*) and *cg20399616 (BCAT1*) have not previously been reported to be associated with adiposity. Therefore, we evaluated these novel CpG sites by checking previously published EWAS for adiposity for having reported these loci. A reported nominal *p* value of < 0.05 at similar direction of effect was considered as significant. We found that *cg07839457* (*NLRC5*) was significantly associated with adiposity in Europeans in visceral adipose tissue (*p* = 2.00E − 04) but not in subcutaneous adipose tissue (*p* = 1) [39]. Both loci were previously reported in an Arab population but neither *cg07839457* (*p* = 1.32E − 01) nor *cg20399616* (*BCAT1*) (*p* = 5.39E − 01) were significantly associated with adiposity in that study [15]. The 15 other EWAS included in the systematic literature search (Additional file 1: File S4) did not report *cg07839457* (*NLRC5*) nor *cg20399616* (*BCAT1*).

### Differentially methylated regions

One DMR was identified overlapping between obesity (FWER = 0.112) and abdominal obesity (FWER = 0.198) (Additional file 1: Figure S10 and Figure S11). This DMR was annotated to chromosome 17 near gene *MIR4520A*. This region contained five CpG sites of the 450k array (*cg13207180*, *cg24686902, cg08103988*, *cg21358336*, *cg08750459*) for obesity and four CpG sites (*cg24686902, cg08103988*, *cg21358336*, *cg08750459*) for abdominal obesity; none of which were significantly associated with any of the anthropometric indices in DMP analyses (FDR ranged between 0.71 and 0.91). A third DMR was associated with obesity only (FWER = 0.124). It contained 13 CpG sites and was annotated to be near gene *RNF39* (Additional file 1: Figure S12).

### Replicated differentially methylated positions reported by previous EWAS

In replication analysis on a subset of 1027 previously reported 450k CpG sites associated with adiposity, we found 15 DMPs associated with BMI at FDR < 0.05 (Table 5). Seven of these (*cg00574958*, *cg25178683*, *cg04927537*, *cg10927968*, *cg11024682*, *cg15871086*, *cg06500161*) were significantly associated with obesity as well (Table 5). DMP *cg00574958* annotated to the 5′ untranslated region of gene *CPT1A*, and DMPs *cg25178683* and *cg04927537* both annotated to the transcription start site of gene *LGALS3BP*, overlapped with the DMPs found in the main epigenome-wide analysis. These DMPs were directionally consistent with the main epigenome-wide analysis. Using a similar approach, we found ten DMPs associated with WC and six with abdominal obesity in the subset of CpG sites from literature (Table 6). The previously mentioned DMPs on *CPT1A* and *LGALS3BP* (*cg00574958*, *cg25178683*, *cg04927537*) were among these six loci and showed directionally consistency with the DMPs found in relation to BMI and obesity. Previous EWAS have reported *cg00574958* (*CPT1A*) in association with both BMI and WC in Arab, European and African-American populations [14–16, 40] while DMPs *cg04927537* and *cg25178683* (*LGALS3BP*) were reported in association with BMI and WC in Arab and African American [14, 15].

**Table 5:** Replicated differentially methylated positions (DMPs) associated with BMI and obesity reported by previous EWAS

					BMI			Obesity		
CpG site	CHR	Position	Nearest gene (distance)^a^	Feature^b^	Beta difference^c^	*p* value^d^	FDR^d^	Beta difference^c^	*p* value^d^	FDR^d^
cg00574958	11	68607622	*CPT1A*	5′ UTR	− 0.0014	1.47E − 07	1.42E − 04	− 0.0189	2.58E − 10	2.49E − 07
cg25178683	17	76976267	*LGALS3BP*	TSS1500	0.0019	1.20E − 06	4.62E − 04	0.0195	8.51E − 05	2.74E − 02
cg04927537	17	76976091	*LGALS3BP*	TSS200	0.0026	1.43E − 06	4.62E − 04	0.0286	6.85E − 06	3.31E − 03
cg10927968	11	1807333	*CTSD* (33351)	IGR	0.0015	2.58E − 05	6.24E − 03	0.0135	2.71E − 04	4.15E − 02
cg17320136	5	10567905	*ANKRD33B*	Body	0.0015	7.47E − 05	1.44E − 02	0.0109	9.60E − 04	1.03E − 01
cg03345232	14	92981121	*RIN3*	Body	0.0022	1.29E − 04	1.78E − 02	0.0193	2.08E − 03	1.66E − 01
cg08726900	16	89550474	*ANKRD11*	5′ UTR	− 0.0029	1.40E − 04	1.78E − 02	− 0.0237	3.85E − 03	2.33E − 01
cg11024682	17	17730094	*SREBF1*	Body	0.0009	1.52E − 04	1.78E − 02	0.0119	1.80E − 04	3.47E − 02
cg15871086	18	56526595	*ZNF532* (− 3466)	IGR	0.0011	1.66E − 04	1.78E − 02	0.0115	1.78E − 04	3.47E − 02
cg18335991	15	74724562	*SEMA7A*	Body	0.0019	2.19E − 04	2.12E − 02	0.0175	2.24E − 03	1.66E − 01
cg07504977	10	102131012	*LINC00263* (− 2321)	IGR	0.0018	2.57E − 04	2.26E − 02	0.0191	8.32E − 04	1.01E − 01
cg27243685	21	43642366	*ABCG1*	Body	0.0005	4.50E − 04	3.46E − 02	0.0054	2.41E − 03	1.66E − 01
cg17439800	1	208056493	*CD34* (− 3390)	IGR	0.0016	4.65E − 04	3.46E − 02	0.0123	2.06E − 02	5.38E − 01
cg06500161	21	43656587	*ABCG1*	Body	0.0005	5.46E − 04	3.77E − 02	0.0077	3.00E − 04	4.15E − 02
cg03546163	6	35654363	*FKBP5*	5′ UTR	0.0019	6.96E − 04	4.49E − 02	0.0181	4.18E − 03	2.38E − 01

^a^CpGs are located in the gene if no distance is indicated. Distance is expressed in kilobase

^b^Based on manifest feature annotation Illumina. IGR, intergenic region

^c^Negative beta values indicate lower DNA methylation (hypo-methylation) in cases compared with controls

^d^ *p* values and FDR corresponding to *M* values. Table is sorted on BMI associated *p* values. All significant hits for both outcomes are included

**Table 6:** Replicated differentially methylated positions (DMPs) associated with WC and abdominal obesity reported by previous EWAS

					WC			Abdominal obesity		
CpG site	CHR	Position	Nearest gene (distance)^a^	Feature^b^	Beta difference^c^	*p* value^d^	FDR^d^	Beta difference^c^	*p* value^d^	FDR^d^
cg00574958	11	68607622	*CPT1A*	5′ UTR	− 0.0007	1.02E − 08	9.87E − 06	−0.0135	1.55E − 08	1.49E − 05
cg04927537	17	76976091	*LGALS3BP*	TSS200	0.0013	6.74E − 08	3.26E − 05	0.0195	4.44E − 05	1.07E − 02
cg25178683	17	76976267	*LGALS3BP*	TSS1500	0.0011	3.96E − 07	1.28E − 04	0.0136	1.54E − 05	7.22E − 03
cg07504977	10	102131012	*LINC00263* (− 2321)	IGR	0.0010	1.60E − 05	3.87E − 03	0.0240	2.24E − 05	7.22E − 03
cg18335991	15	74724562	*SEMA7A*	Body	0.0009	2.22E − 05	4.30E − 03	0.0147	3.75E − 04	7.24E − 02
cg27243685	21	43642366	*ABCG1*	Body	0.0004	7.58E − 05	1.22E − 02	0.0037	1.72E − 02	3.77E − 01
cg10927968	11	1807333	*CTSD*	IGR	0.0007	9.42E − 05	1.30E − 02	0.0082	1.27E − 02	3.77E − 01
cg06500161	21	43656587	*ABCG1*	Body	0.0003	2.15E − 04	2.60E − 02	0.0033	3.47E − 03	2.76E − 01
cg13123009	6	31681882	*LY6G6E*	TSS200	0.0004	3.22E − 04	3.46E − 02	0.0074	3.65E − 03	2.76E − 01
cg17439800	1	208056493	*CD34*	IGR	0.0006	5.09E − 04	4.92E − 02	0.0205	5.83E − 04	9.39E − 02

^a^CpGs are located in the gene if no distance is indicated. Distance is expressed in kilobase

^b^Based on manifest feature annotation Illumina. IGR, intergenic region

^c^Negative beta values indicate lower DNA methylation (hypo-methylation) in cases compared with controls

^d^ *p* values and FDR corresponding to *M* values. Table is sorted on WC associated *p* values. All significant hits for both outcomes are included

### Replicated differentially methylated regions reported in non-African populations

None of the EWAS selected through our systematic literature search identified DMRs using similar methods. One study identified regions based on mean methylation index of CpG sites within a predefined region [41]. These five regions did not overlap with the DMRs identified in the present study.

### Pathway analyses

Pathway analyses resulted in 67 enriched GO categories in relation to BMI and 61 in relation to WC. Twenty-six of these BMI GO categories contained either one or more DMPs associated with BMI and obesity (Additional file 1: Table S13-A). Pathways were mostly involved in immune function, cell signalling and regulation. Out of the WC pathways, five pathways contained the DMP annotated to gene *CPT1A* associated with WC and abdominal obesity (Additional file 1: Table S13-B). These pathways were related to peptide transport and regulation.

## Discussion

In the present study, we report the first EWAS for adiposity among sub-Saharan Africans using a sample of Ghanaians from the RODAM study. We found DMP *cg00574958* annotated to gene *CPT1A* to be significantly associated with BMI, WC, obesity and abdominal obesity. DMPs *cg07839457* and *cg20399616*, annotated to genes *NLRC5* and *BCAT1*, respectively, were associated with BMI, WC and obesity but not with abdominal obesity. These two DMPs are novel adiposity loci as they have not previously been reported in any EWAS for adiposity.

Direct measurement of adiposity, for example by computed tomography, is rarely feasible in the field epidemiological studies. Therefore, we evaluated two commonly used epidemiological indicators of adiposity, namely BMI and WC both continuous and dichotomised. BMI is an index for generalized adiposity or overall body size and is most widely used. WC is thought to better capture the metabolic complications of adiposity [42]. WC has been found to correlate well with abdominal imaging [43] and is associated with metabolic diseases such as type 2 diabetes [44]. Fourteen of the 41 DMPs (34%) overlapped between BMI and WC (Fig. 2) and were directionally consistent. This is considerably lower than the 74% DMP overlap between BMI and WC previously reported in African Americans [14]. A possible explanation for this discrepancy is that BMI and WC identify different individuals at risk in our Ghanaian population as is shown by the fact that there were 132 more with abdominal obesity than with general adiposity in this study sample. Previous studies have shown differences in fat distribution between African descent and European populations characterized by less abdominal fat in Africans [45]. A previous study in Ghanaians found poor discriminative ability of BMI for type 2 diabetes compared with indices for abdominal obesity [46]. Potentially, increases in WC are more pronounced in this population than increases in BMI, which might underlie the high type 2 diabetes burden in this population [3]. Our finding that in Ghanaians, the DMP annotated to gene *CPT1A* was associated strongly with abdominal obesity suggests the need to explore the role of *CPT1A* variation in DNA methylation related to increases in WC and increased risk for type 2 diabetes.

*CPT1A*, or carnitine palmitoyltransferase 1A, has been reported in relation to BMI, obesity and weight gain in both GWAS and EWAS of multiple populations [14–16]. The DMP *cg00574958* is annotated to the 5′ untranslated region, a region important in regulation, of the *CPT1A* gene. The *CPT1A* gene codes for the carnitine palmitoyltransferase 1 enzyme, which is crucial in fatty acid beta-oxidation [47]. Beta oxidation is a catabolic process in fatty acid metabolism. We observed hypo-methylation at *cg00574958* for higher BMI and WC. A previous study has shown that higher DNA methylation at *cg00574958* correlated negatively with *CPT1A* expression in blood [48]. Hypo-methylation at *cg00574958* could thus be involved in increased expression of *CPT1A* and subsequent increased enzyme production. This suggests more beta oxidation is taking place in those with more adiposity. Hypo-methylation at *cg00574958* could be the result of the high fat mass which requires a more active fatty acid metabolism. This is in line with previous studies that have reported modifications in DNA methylation as a result of changes in lipid metabolism due to adiposity rather than as a cause [49]. In our study population, *CPT1A* attributed to 6.1% of the variance in obesity and 5.6% of the variance in abdominal obesity.

*NLRC5* (NLR family CARD domain containing 5) and *BCAT1* (branched chain amino acid transaminase 1) are novel adiposity candidate loci as they have not been reported in previous EWAS. In pathway analyses (Additional file 1: Table S13-A and S13-B), we found that *NLR5* is involved in multiple immune response pathways. The *NLRC5* gene regulates the expression of major histocompatibility complex (MHC) class I genes [50]. MHC class I molecules play an important role in immune function, in particular in the activation of CD8+ T cells against viral infections. CD8+ T cells also play an essential role in onset of adipose tissue inflammation [51]. Other studies have shown that *NLRC5* can be upregulated in response to inflammatory stimulation [52]. Excess adipose tissue has regularly been associated with induced inflammation [53]. DMP *cg07839457* located 1.5 kb upstream of the transcription start site (TSS) of *NLRC5* was found in our study to be hyper-methylated for higher BMI and WC, and 7.1% hyper-methylated in those with obesity compared with those without (Tables 2 and 3). Hyper-methylation of TSS regions is in general associated with long-term silencing [54] which could imply lower expression of *NLRC5* gene in adiposity. Functional studies are needed to investigate the role of the *NLRC* gene in adiposity among sub-Saharan Africans.

DMP *cg20399616* annotated to gene *BCAT1* was hypo-methylated in relation to BMI, WC and obesity. *BCAT1* encodes for a protein that catalyses catabolism of amino acids [55]. This gene has shown very low methylation levels in blood in patients with colorectal cancer [56] and is being explored to be used as biomarker in colorectal cancer assays [57]. *BCAT1* has also been found hypo-methylated in other cancer types such as ovarian cancer [58]. The relative risk of several types of cancer has been found higher in obese compared with non-obese [59]. Our data suggest that this could potentially be mediated by DNA methylation variations caused by adiposity.

Little is known about the translatability of EWAS findings among Europeans to sub-Saharan Africans. The few EWAS conducted in non-European populations identified novel loci involved in adiposity in African Americans and Arabs [14, 15, 18]. These studies found transferability between European and other populations to be relatively low. Out of 47 EWAS associations reported in Europeans, seven were replicated in Arab populations and showed heterogeneity in effects between both populations for all loci except *CPT1A* [15]. Transferability of findings among African Americans to Europeans was somewhat higher: 62% of epigenome-wide DMPs in African Americans were replicated in Europeans [14]. The top DMPs in replication again included *CPT1A*. In the present study, we found transferability with BMI for 15 previously reported DMPs and with WC for 10 previously reported DMPs on adiposity. These loci, in particular the DMP annotated to *CPT1A*, may play a role in adiposity across populations.

A potential limitation to our study is the use of blood samples as DNA methylation is tissue specific. However, several studies have shown moderate to strong correlations between blood tissue DNA methylation and other tissue types such as subcutaneous fat [49]. Specifically, the methylation level of gene *CPT1A* showed cross-tissue agreement between blood and adipose tissue in previous studies [14]. Another limitation to our study is that due to the cross-sectional nature of our study, we cannot make statements about causality. A recent meta-analyses showed that changes in DNA methylation were rather the consequence of adiposity than the cause [49]. Evidence from intervention studies strengthens this notion. For example, a study on weight loss found 432 DMPs before dietary intervention of which 15 DMPs remained at the end of the intervention when participants had lost weight [60]. Other studies have shown that DNA methylation changes induced by a high-fat diet can be partly reversed after a 6–8-week washout period [61]. The DMPs we identified also seemed the result of adiposity given the biological plausibility of the genes involved. A possible limitation of the present study is the possibility that some of the hits reported are due to genetic (e.g. SNPs) rather than epigenetic variation. Unfortunately, we do not yet have GWAS SNP data on the RODAM cohort that would allow us directly test this hypothesis. Furthermore, we observed some inflation in evaluation of QQ plots (Additional file 1: File S2). However, methods for inflation correction (Additional file 1: File S3) did not substantially improve model fitting nor did it alter our results regarding the DMPs identified. The results of this EWAS should be confirmed in follow-up studies to disentangle the etiological role of DMPs we identified. These follow-up studies should include translational research as well as a characterization of the role of environment versus genetics in observed DNA methylation findings.

## Conclusions

In conclusion, we report the first EWAS on adiposity among sub-Saharan Africans. Notably, we found three DMPs or loci (*CPT1A*, *NLRC5* and *BCAT1*) that showed epigenome-wide significance with both obesity and abdominal obesity. Translational studies and longitudinal study designs are warranted to determine the role of these DNA methylation variants in the high burden of adiposity among sub-Saharan Africans.

## Additional file

Additional file 1: Additional methods, quality control, sensitivity analyses and supplementary results. (ZIP 1243 kb)

## Abbreviations

BMI: Body mass index
CpG site: Potentially methylated site on the genome where a cytosine nucleotide is followed by guanine
DMP: Differentially methylated position
DMR: Differentially methylated region
EWAS: Epigenome-wide association studies
FDR: False discovery rate
FWER: Family-wise error rate
PCA: Principal component analysis
RODAM: Research on Obesity and Diabetes among African Migrants
WC: Waist circumference
