# Supplementary material for: An epigenome-wide association study in whole blood of measures of adiposity among Ghanaians: the RODAM study
Source: Clin Epigenetics. 2017 Sep 21;9:103. doi: 10.1186/s13148-017-0403-x (PMC5609006; doi:10.1186/s13148-017-0403-x)
Supplement: Additional file 1: — Additional methods, quality control, sensitivity analyses and supplementary results. (ZIP 1243 kb) [file 13148_2017_403_MOESM1_ESM.zip › 13148_2017_403_MOESM1_ESM/Supplementary File S5.pdf]

Candidate probe (450k)	chr	pos	UCSC_RefGene_Name
cg08122070	chr1	1689610	NADK
cg18269141	chr1	2063966	PRKCZ;PRKCZ;PRKCZ
cg00175702	chr1	2109388	PRKCZ;PRKCZ;PRKCZ
cg22229039	chr1	7842159	
cg04993279	chr1	8940460	
cg07499142	chr1	9788715	PIK3CD
cg05599320	chr1	9883032	CLSTN1;CLSTN1
cg06829969	chr1	10460205	PGD
cg17514528	chr1	11862907	MTHFR
cg19620994	chr1	12774904	AADACL3;AADACL3
cg07708453	chr1	14032034	PRDM2;PRDM2;PRDM2;PRDM2;PRDM2
cg03586820	chr1	16679780	FBXO42
cg26227957	chr1	19547285	KIAA0090
cg26561681	chr1	21376633	EIF4G3
cg16603916	chr1	21615863	ECE1;ECE1;ECE1
cg18660064	chr1	23504632	
cg15961455	chr1	23590501	
cg11078090	chr1	23878540	
cg18150584	chr1	23887816	ID3
cg08540100	chr1	23888986	
cg24445316	chr1	23889092	
cg01519464	chr1	24861818	RCAN3
cg26692749	chr1	24861919	RCAN3
cg11377047	chr1	26881009	MIR1976;RPS6KA1;RPS6KA1
cg03604774	chr1	27849102	
cg12484113	chr1	27898757	AHDC1
cg23840027	chr1	29225533	EPB41;EPB41;EPB41;EPB41;EPB41
cg19510565	chr1	31217240	LAPTM5
cg24401262	chr1	31956405	
cg11683242	chr1	32716557	LCK
cg23202722	chr1	33793808	PHC2;PHC2
cg04011995	chr1	41326317	
cg25463399	chr1	43820383	
cg00754357	chr1	53019727	ZCCHC11;ZCCHC11;ZCCHC11
cg06358171	chr1	54822008	SSBP3;SSBP3;SSBP3
cg10266490	chr1	55013709	ACOT11;ACOT11
cg17901584	chr1	55353706	DHCR24
cg04084354	chr1	56721795	
cg10220544	chr1	60171660	FGGY;FGGY
cg12262372	chr1	61542211	NFIA
cg08036553	chr1	64649805	
cg22945413	chr1	65399413	JAK1
cg18854765	chr1	74665362	LRRIQ3;TNNI3K;FPGT
cg09935388	chr1	92947588	GFI1;GFI1;GFI1
cg06788267	chr1	95258162	
cg26063904	chr1	101313199	
cg24323958	chr1	108741884	SLC25A24
cg06756164	chr1	109490978	CLCC1;CLCC1
cg16140253	chr1	110166563	AMPD2;AMPD2;AMPD2
cg24056365	chr1	110834615	
cg21419003	chr1	112048205	ADORA3;ADORA3;ADORA3
cg00960147	chr1	117052153	
cg14476101	chr1	120255992	PHGDH
cg02976588	chr1	150135546	
cg03763518	chr1	150245044	C1orf54
cg00944785	chr1	150825365	ARNT;ARNT;ARNT
cg24678869	chr1	153919638	DENND4B
cg04488111	chr1	154897518	PMVK
cg16097041	chr1	154965544	FLAD1;LENEP;FLAD1
cg23998749	chr1	154968781	
cg16374333	chr1	157103641	ETV3;ETV3
cg07196514	chr1	158984321	IFI16
cg07202479	chr1	159174162	DARC;DARC
cg05954830	chr1	159838031	
cg17833106	chr1	161060000	PVRL4
cg08894273	chr1	161109487	
cg13178361	chr1	162532502	UAP1

cg04869770	chr1	164561550	PBX1
cg23114435	chr1	167442612	CD247;CD247
cg09554443	chr1	167487762	CD247;CD247;CD247;CD247
cg13501279	chr1	167523555	CREG1
cg04846203	chr1	167690438	MPZL1;MPZL1;MPZL1
cg01565130	chr1	167690726	MPZL1;MPZL1;MPZL1
cg07948875	chr1	169187004	NME7;NME7
cg16696002	chr1	172189728	DNM3;DNM3
cg23933289	chr1	178998656	FAM20B
cg24448340	chr1	179921042	
cg09472600	chr1	183537770	NCF2;NCF2
cg04231677	chr1	184808004	FAM129A
cg25408950	chr1	198290080	NEK7
cg26257082	chr1	198906494	
cg07699546	chr1	201790194	NAV1;NAV1
cg07104135	chr1	201799785	IPO9
cg06650246	chr1	206897270	MAPKAPK2;MAPKAPK2
cg14180511	chr1	206946187	IL10
cg14856606	chr1	207277547	C4BPA
cg17439800	chr1	208056493	
cg10721755	chr1	211547951	TRAF5;TRAF5;TRAF5
cg15134583	chr1	224015075	TP53BP2;TP53BP2
cg19867250	chr1	225648715	
cg10826999	chr1	225862288	
cg12221970	chr1	226884832	ITPKB
cg23230158	chr1	230260837	GALNT2
cg26303777	chr1	230311676	GALNT2
cg16647844	chr1	232903627	
cg12869659	chr1	244213618	ZNF238
cg00967073	chr1	244443687	
cg00864945	chr1	247291374	
cg03466998	chr1	247580074	NLRP3;NLRP3;NLRP3;NLRP3;NLRP3
cg11422335	chr1	247611517	NLRP3;NLRP3;NLRP3;NLRP3;NLRP3
cg21655790	chr1		LEPR
cg01462537	chr10	2992189	
cg27367170	chr10	5488628	NET1;NET1;NET1
cg26105232	chr10	6105656	IL2RA
cg08243626	chr10	6442501	
cg05103064	chr10	7311500	SFMBT2
cg06230736	chr10	8096650	FLJ45983;GATA3;GATA3;FLJ45983
cg05392293	chr10	13911331	FRMD4A
cg26852723	chr10	15356129	FAM171A1
cg10368935	chr10	18240316	SLC39A12;SLC39A12
cg25181170	chr10	25162298	PRTFDC1
cg11316887	chr10	29924694	SVIL;SVIL
cg19223064	chr10	30351259	
cg08764162	chr10	31147088	ZNF438;ZNF438;ZNF438;ZNF438;ZNF438;ZNF438;ZNF438;ZNF438
cg06528823	chr10	32303107	KIF5B
cg02561009	chr10	43391363	
cg19921651	chr10	44494208	
cg00899659	chr10	45495971	C10orf25;ZNF22
cg10501360	chr10	49671665	ARHGAP22
cg20166027	chr10	51576452	NCOA4;NCOA4;NCOA4;NCOA4;NCOA4;NCOA4
cg25164589	chr10	63540024	
cg18980410	chr10	63764155	ARID5B
cg18522931	chr10	63776828	ARID5B
cg03201337	chr10	69609995	
cg27074971	chr10	71072149	HK1;HK1;HK1
cg20283498	chr10	73387917	CDH23;CDH23
cg04304802	chr10	73499965	CDH23
cg26033520	chr10	74004071	
cg10919111	chr10	74075783	
cg20205477	chr10	74113178	DNAJB12;DNAJB12
cg13852284	chr10	77548353	C10orf11
cg13424302	chr10	80516893	
cg17940587	chr10	89292231	MINPP1
cg13404688	chr10	89876324	
cg14349937	chr10	90093521	RNLS;RNLS

cg03249630	chr10	90611782	ANKRD22
cg01561719	chr10	90611855	ANKRD22
cg26478401	chr10	90749920	FAS;FAS;FAS;FAS;FAS;FAS;FAS;ACTA2
cg20180364	chr10	94448532	HHEX
cg13471990	chr10	97515222	ENTPD1;ENTPD1;ENTPD1;ENTPD1;ENTPD1;ENTPD1;ENTPD1
cg13496098	chr10	97666894	C10orf131
cg12182708	chr10	99223744	MMS19
cg08181251	chr10	99443455	AVPI1
cg17044311	chr10	101542983	ABCC2
cg07504977	chr10	102131012	
cg18390025	chr10	103986736	ELOVL3
cg02909372	chr10	104193735	CUEDC2
cg15219811	chr10	104259328	ACTR1A
cg17782974	chr10	104406990	TRIM8
cg04774043	chr10	104498430	SFXN2
cg18729704	chr10	105159484	PDCD11
cg17841267	chr10	112117449	
cg00066854	chr10	113987376	
cg00750430	chr10	118654150	KIAA1598;KIAA1598
cg06474225	chr10	124228770	HTRA1
cg11803859	chr10	125770124	CHST15
cg04726013	chr10	126223236	LHPP;LHPP
cg14683065	chr10	134149184	LRRC27;LRRC27;LRRC27;LRRC27;LRRC27
cg06760238	chr10	134400036	INPP5A
cg09163720	chr10	134400506	INPP5A
cg17444479	chr10	134429869	INPP5A
cg16284674	chr10	135123337	ZNF511;TUBGCP2
cg06072036	chr11	822402	PNPLA2
cg23240927	chr11	1320497	TOLLIP
cg22680424	chr11	1778547	HCCA2;CTSD
cg10927968	chr11	1807333	
cg23314972	chr11	1977533	MRPL23
cg12949760	chr11	2542862	KCNQ1;KCNQ1
cg06294475	chr11	2698623	KCNQ1;KCNQ1OT1;KCNQ1
cg20651018	chr11	3035856	CARS;CARS;CARS;CARS
cg10611016	chr11	6225759	C11orf42
cg14838970	chr11	7533817	PPFIBP2
cg15228694	chr11	7692131	CYB5R2
cg12165551	chr11	8385712	
cg09976669	chr11	9738663	SWAP70
cg24435741	chr11	10471371	AMPD3
cg05181279	chr11	11982794	
cg07702424	chr11	12306515	
cg15312943	chr11	17843855	SERGEF
cg15446391	chr11	32452370	WT1;WT1;WT1;WT1
cg04225088	chr11	33278186	HIPK3;HIPK3
cg05501357	chr11	33308269	HIPK3;HIPK3
cg25600606	chr11	33308345	HIPK3;HIPK3
cg04036920	chr11	33562503	C11orf41
cg15356966	chr11	33913187	LMO2
cg11822932	chr11	33913716	LMO2;LMO2
cg07136133	chr11	36422377	PRR5L;PRR5L;PRR5L;PRR5L
cg02211741	chr11	46383924	DGKZ;DGKZ;DGKZ;DGKZ
cg17580616	chr11	47350136	MADD;MADD;MADD;MADD;MADD;MADD;MADD;MADD;MADD;MADD
cg10426076	chr11	57158282	PRG2
cg21272996	chr11	57529255	CTNND1;CTNND1
cg19210276	chr11	57529465	CTNND1;CTNND1
cg20018806	chr11	59633874	TCN1
cg00009088	chr11	60930188	VPS37C
cg24422316	chr11	60930346	VPS37C
cg20518446	chr11	62315034	AHNAK;AHNAK
cg07029024	chr11	65249342	
cg10142520	chr11	65344604	EHBP1L1
cg22151881	chr11	68082621	LRP5
cg00574958	chr11	68607622	CPT1A;CPT1A
cg00242341	chr11	72447419	ARAP1
cg18115721	chr11	73567838	MRPL48
cg20281309	chr11	78127618	GAB2

cg11660018	chr11	86510915	PRSS23
cg23502204	chr11	87905295	RAB38
cg01568784	chr11	87908783	RAB38
cg15412815	chr11	93271088	C11orf75
cg02856190	chr11	94282491	FUT4;FUT4
cg23018873	chr11	94501467	AMOTL1
cg24941342	chr11	95093809	
cg07530172	chr11	95431373	
cg15126862	chr11	100696271	FLJ32810
cg14191024	chr11	110070509	
cg03304763	chr11	112037427	TEX12
cg10953410	chr11	113846918	HTR3A;HTR3A;HTR3A
cg22130673	chr11	114031817	ZBTB16;ZBTB16
cg19069882	chr11	118123243	MPZL3
cg24612198	chr11	118175631	CD3E
cg24841244	chr11	118213330	CD3D;CD3D;CD3D;CD3D
cg11474701	chr11	121193898	
cg03463948	chr11	122612858	UBASH3B
cg24559261	chr11	126177731	DCPS
cg23855879	chr11	129684323	TMEM45B
cg13255542	chr11	134120785	THYN1;THYN1;THYN1;THYN1;THYN1
cg19037107	chr11	134126323	ACAD8
cg21648069	chr12	1157718	ERC1;ERC1;ERC1;ERC1;ERC1
cg16303353	chr12	2393684	CACNA1C;CACNA1C;CACNA1C
cg15719903	chr12	6570167	TAPBPL
cg13298466	chr12	6658164	IFFO1;IFFO1;IFFO1;IFFO1
cg24437859	chr12	7066614	PTPN6;PTPN6;PTPN6
cg07052231	chr12	7363540	PEX5;PEX5;PEX5;PEX5;PEX5
cg09142829	chr12	8276482	CLEC4A;CLEC4A;CLEC4A;CLEC4A
cg21685770	chr12	10324918	OLR1
cg14693090	chr12	11899281	ETV6
cg16310192	chr12	11966066	ETV6
cg06530347	chr12	25148943	C12orf77
cg01023672	chr12	47477223	
cg01923089	chr12	49627746	
cg06712013	chr12	49759545	SPATS2
cg24637417	chr12	51636921	DAZAP2;DAZAP2;DAZAP2;DAZAP2;DAZAP2;DAZAP2
cg09802818	chr12	52604609	LOC283404
cg08730245	chr12	53902893	NPFF
cg09303642	chr12	54690818	NFE2;NFE2
cg01324474	chr12	54758545	GPR84
cg10056728	chr12	56497073	PA2G4;ERBB3
cg26545245	chr12	57382283	
cg06317209	chr12	58210878	AVIL
cg12707346	chr12	64960957	
cg12488187	chr12	65671664	MSRB3;MSRB3
cg26244575	chr12	76354015	
cg06655216	chr12	89619329	
cg02806156	chr12	89728752	
cg01305421	chr12	102874286	IGF1;IGF1;IGF1;IGF1;IGF1;IGF1
cg13135241	chr12	104765292	
cg00608661	chr12	105065028	CHST11
cg12053762	chr12	107167543	RIC8B
cg00859441	chr12	113799660	PLBD2;PLBD2
cg09887059	chr12	113916664	
cg00355804	chr12	117103567	
cg17627898	chr12	118782453	TAOK3
cg07545743	chr12	121409957	C12orf27
cg16942632	chr12	121726240	CAMKK2;CAMKK2;CAMKK2;CAMKK2;CAMKK2;CAMKK2;CAMKK2
cg15695155	chr12	121973871	KDM2B;KDM2B
cg26995224	chr12	121974146	KDM2B;KDM2B
cg13708645	chr12	121974305	KDM2B;KDM2B
cg10857250	chr12	122986080	ZCCHC8
cg15545247	chr12	123201372	GPR109B;GPR109B
cg24967811	chr12	123503709	PITPNM2
cg15258936	chr12	124421582	CCDC92
cg22700848	chr12	124990942	NCOR2;NCOR2
cg19717347	chr12	129304956	SLC15A4

cg21046080	chr12	131506092	GPR133
cg19843457	chr12	132288611	
cg07920381	chr12	132303685	
cg15235987	chr12	132469937	EP400
cg22274414	chr13	19756318	TUBA3C
cg07879785	chr13	21095712	CRYL1
cg20227511	chr13	28670602	FLT3
cg20956594	chr13	29202889	
cg25936902	chr13	30982971	
cg16396191	chr13	42184765	KIAA0564
cg15894389	chr13	47470857	HTR2A;HTR2A;HTR2A
cg02250787	chr13	47470989	HTR2A;HTR2A;HTR2A;HTR2A
cg09361691	chr13	47471169	HTR2A;HTR2A
cg11514288	chr13	47471197	HTR2A;HTR2A
cg27068143	chr13	47471264	HTR2A;HTR2A
cg10323433	chr13	47471562	HTR2A;HTR2A
cg09798090	chr13	47472140	HTR2A;HTR2A
cg15692052	chr13	47472250	HTR2A;HTR2A
cg11093142	chr13	49075016	RCBTB2
cg18546006	chr13	50569769	TRIM13;TRIM13;TRIM13;TRIM13;DLEU2
cg09145126	chr13	50570018	TRIM13;TRIM13;TRIM13;TRIM13;DLEU2
cg26776551	chr13	51944507	INTS6;INTS6
cg09855140	chr13	100004097	UBAC2;FKSG29;UBAC2;UBAC2
cg09646392	chr13	108921052	TNFSF13B;TNFSF13B
cg08400494	chr13	111318490	CARS2
cg18535410	chr13	113793268	F10
cg06380691	chr13	114828264	RASA3
cg07300408	chr14	21058360	RNASE12;RNASE11;RNASE12;RNASE11
cg24162781	chr14	21089025	
cg07525077	chr14	21359943	RNASE3
cg01797899	chr14	22978195	
cg03035162	chr14	22993190	
cg09232358	chr14	23015657	
cg04756491	chr14	23385384	RBM23;RBM23;RBM23
cg15377871	chr14	23588325	CEBPE;CEBPE
cg13277939	chr14	39735211	CTAGE5;CTAGE5;CTAGE5;CTAGE5
cg21502048	chr14	52780158	PTGER2
cg21814550	chr14	53170147	
cg01860774	chr14	64969374	ZBTB25
cg01424562	chr14	69256677	ZFP36L1
cg15935770	chr14	70160160	KIAA0247
cg02132667	chr14	80957817	
cg10825315	chr14	81425912	TSHR;TSHR;TSHR
cg11168614	chr14	87179368	
cg15625636	chr14	88472437	GPR65
cg03345232	chr14	92981121	RIN3
cg03562528	chr14	94423943	ASB2
cg23661721	chr14	95991371	
cg08253808	chr14	102676957	WDR20
cg16364675	chr14	103096221	RCOR1
cg19711268	chr14	103367858	TRAF3;TRAF3;TRAF3
cg14191466	chr14	103440432	CDC42BPB
cg18568335	chr14	105792505	PACS2;PACS2
cg23594345	chr14	106329607	
cg26033681	chr15	25069376	SNRPN;SNRPN;SNRPN
cg09958402	chr15	26044116	ATP10A
cg07814318	chr15	31624584	KLF13
cg04857231	chr15	42782520	
cg10327976	chr15	42843303	HAUS2;HAUS2
cg15159104	chr15	43809865	MAP1A;MAP1A
cg23671196	chr15	50405520	ATP8B4
cg02253142	chr15	52048211	TMOD2;TMOD2
cg14120436	chr15	52483498	GNB5
cg06192883	chr15	52554171	MYO5C
cg18812353	chr15	56385430	RFX7
cg11098259	chr15	58430391	AQP9
cg11952340	chr15	63814440	USP3
cg10207553	chr15	65277977	SPG21;SPG21;SPG21

cg02328326	chr15	68038930	MAP2K5;MAP2K5
cg26575166	chr15	70307202	
cg24405567	chr15	70787565	
cg24996282	chr15	74692822	
cg18335991	chr15	74724562	SEMA7A;SEMA7A;SEMA7A
cg02119938	chr15	78505051	ACSBG1
cg22821300	chr15	79296585	RASGRF1;RASGRF1;RASGRF1
cg21840434	chr15	79297210	RASGRF1;RASGRF1;RASGRF1;RASGRF1
cg11592786	chr15	89533581	
cg13460556	chr15	89709638	ABHD2;ABHD2
cg18568872	chr15	90606494	ZNF710
cg09397246	chr15	91427361	FES;FES;FES;FES
cg27614723	chr15	92399897	SLCO3A1;SLCO3A1
cg14138540	chr15	93571988	
cg23054181	chr15	99048945	FAM169B
cg23047825	chr15	99317340	IGF1R
cg02579959	chr15	100890963	FLJ42289;FLJ42289
cg24595580	chr15	100890996	FLJ42289;FLJ42289
cg12042714	chr15	101715747	
cg19997662	chr15	101784653	CHSY1
cg09050670	chr16	1521617	CLCN7;CLCN7
cg07611933	chr16	2773245	
cg26680760	chr16	3155880	
cg08494738	chr16	4369512	
cg01678580	chr16	4674018	MGRN1;MGRN1;MGRN1;MGRN1
cg02435083	chr16	8943436	
cg26434370	chr16	10909361	FAM18A
cg08493063	chr16	11330934	
cg06946797	chr16	11422409	
cg03308839	chr16	15797297	NDE1;NDE1;MYH11;MYH11;MYH11;MYH11
cg04885396	chr16	16067359	ABCC1;ABCC1;ABCC1;ABCC1;ABCC1
cg09109383	chr16	16083164	ABCC1;ABCC1;ABCC1;ABCC1;ABCC1
cg04986899	chr16	17553784	XYLT1
cg06129210	chr16	17565129	XYLT1
cg04695882	chr16	20756332	
cg08224563	chr16	20916305	LYRM1;LYRM1;LYRM1
cg07079231	chr16	21169331	DNAH3;TMEM159
cg05240166	chr16	21171239	TMEM159;DNAH3
cg26562691	chr16	23850404	PRKCB;PRKCB
cg02656594	chr16	27412496	IL21R
cg26663590	chr16	28959310	
cg16519923	chr16	30485810	ITGAL;ITGAL
cg01014262	chr16	48533930	
cg01701649	chr16	48589954	N4BP1
cg07468327	chr16	54170217	
cg00863378	chr16	56549757	BBS2
cg04304036	chr16	68334619	SLC7A6;SLC7A6;SLC7A6OS
cg06285909	chr16	69098945	TMCO7
cg04703221	chr16	69967063	WWP2;MIR140;WWP2
cg01389386	chr16	70471055	ST3GAL2
cg16422316	chr16	71844250	AP1G1;AP1G1
cg05083852	chr16	81480610	CMIP
cg03543954	chr16	85116335	KIAA0513
cg03848483	chr16	85969392	
cg01787084	chr16	87371097	FBXO31;FBXO31
cg06928952	chr16	87736670	LOC100129637
cg00153919	chr16	88859944	
cg08551036	chr16	89219820	ACSF3;ACSF3;ACSF3
cg03819692	chr16	89225639	C16orf81
cg05453411	chr16	89300123	
cg08721324	chr16	89384602	ANKRD11
cg08726900	chr16	89550474	ANKRD11
cg13396713	chr17	505019	VPS53;VPS53
cg18317439	chr17	643637	FAM57A
cg01438467	chr17	1510080	SLC43A2
cg17141696	chr17	1680805	SERPINF1
cg12789448	chr17	1979495	SMG6;SMG6
cg09664445	chr17	2612406	KIAA0664

cg22044342	chr17	3819339	P2RX1
cg25031824	chr17	3819363	P2RX1
cg00277591	chr17	4079652	ANKFY1
cg23387401	chr17	4582204	PELP1
cg13221924	chr17	6495080	KIAA0753
cg13194425	chr17	7341936	FGF11
cg05872923	chr17	7461260	TNFSF13;TNFSF13;TNFSF12-TNFSF13;TNFSF13
cg05514680	chr17	7461556	TNFSF12-TNFSF13;TNFSF13;TNFSF13;TNFSF13
cg00582663	chr17	7815834	CHD3;CHD3;CHD3
cg02067584	chr17	8094552	C17orf59
cg05460226	chr17	8804279	PIK3R5;PIK3R5
cg15252429	chr17	9676243	DHRS7C
cg08826152	chr17	15869607	ADORA2B
cg19447962	chr17	17628656	RAI1
cg11024682	chr17	17730094	SREBF1;SREBF1
cg17126924	chr17	25773641	
cg05475440	chr17	26452975	NLK
cg01347228	chr17	29297391	RNF135;RNF135
cg18483549	chr17	30842946	MYO1D
cg18067859	chr17	33776345	SLFN13
cg02380585	chr17	33776683	SLFN13
cg14834285	chr17	33897374	
cg08542715	chr17	34217960	
cg17939040	chr17	35503956	ACACA;ACACA;ACACA;ACACA;ACACA
cg15445000	chr17	37608096	MED1
cg04272820	chr17	38228538	THRA;THRA
cg26854588	chr17	38440015	
cg13274938	chr17	38493822	RARA;RARA;RARA
cg01455550	chr17	39143980	KRT40
cg13053608	chr17	40345673	GHDC;GHDC;GHDC;GHDC
cg08857797	chr17	40927699	VPS25
cg10499832	chr17	41450195	LOC100130581
cg03078551	chr17	41656298	
cg18219562	chr17	41773643	
cg14039779	chr17	41857714	DUSP3;C17orf105
cg19601328	chr17	43368729	MAP3K14
cg19104015	chr17	46646043	HOXB3
cg01351315	chr17	46667737	LOC404266;LOC404266
cg00711072	chr17	46669489	LOC404266;HOXB5;LOC404266;LOC404266;LOC404266
cg12744859	chr17	46669492	LOC404266;HOXB5;LOC404266;LOC404266;LOC404266
cg26664457	chr17	47394423	ZNF652;ZNF652
cg20925233	chr17	47661136	NXPH3
cg04582295	chr17	48242070	SGCA;SGCA
cg05916684	chr17	49008322	
cg07493197	chr17	53573969	
cg08562099	chr17	55389809	MSI2;MSI2
cg05844827	chr17	55502491	MSI2;MSI2
cg21139312	chr17	55663225	MSI2;MSI2
cg13984330	chr17	58637589	
cg15723222	chr17	59222476	BCAS3;BCAS3
cg13243168	chr17	61915833	SMARCD2;SMARCD2
cg17463083	chr17	62153284	ERN1
cg05467716	chr17	62774079	LOC146880
cg06405341	chr17	63535223	AXIN2
cg03469804	chr17	72732432	RAB37;RAB37;RAB37;RAB37
cg08661469	chr17	72732823	RAB37;RAB37;RAB37;RAB37
cg00900735	chr17	73316505	GRB2;GRB2
cg21486834	chr17	74477542	RHBDF2;RHBDF2
cg03330678	chr17	75316233	SEPT9;SEPT9;SEPT9;SEPT9
cg18181703	chr17	76354621	SOCS3
cg15010903	chr17	76850256	TIMP2
cg01502428	chr17	76850266	TIMP2
cg04927537	chr17	76976091	LGALS3BP
cg25178683	chr17	76976267	LGALS3BP
cg20402747	chr17	77954963	TBC1D16
cg01432609	chr17	78638554	RPTOR;RPTOR
cg11222173	chr17	78748019	RPTOR;RPTOR
cg13618516	chr17	79129078	AATK

cg25616968	chr17	79416203	BAHCC1
cg27200257	chr17	79816678	P4HB
cg19573490	chr17	79870317	PCYT2;SIRT7
cg05655915	chr17	80415420	NARF;NARF;NARF;NARF
cg11690666	chr17	80415469	NARF;NARF;NARF;NARF
cg02116768	chr17	80545322	FOXK2
cg13424393	chr17	80569923	
cg25197500	chr17	80581805	WDR45L
cg03778909	chr17	80833393	TBCD
cg13304638	chr17	80834089	TBCD
cg07139527	chr17	80969525	B3GNTL1
cg13461118	chr18	9929439	VAPA;VAPA
cg07453440	chr18	11948154	
cg18517540	chr18	32446543	DTNA;DTNA;DTNA;DTNA;DTNA;DTNA;DTNA
cg27113059	chr18	47088248	LIPG
cg21616935	chr18	47794231	CCDC11
cg18934187	chr18	51882189	STARD6
cg15871086	chr18	56526595	
cg17251713	chr18	61443038	SERPINB7;SERPINB7
cg22095490	chr18	77560089	
cg11768182	chr19	691833	PRSSL1
cg24347562	chr19	839678	PRTN3
cg07239938	chr19	852813	ELANE
cg07573872	chr19	1126342	SBNO2;SBNO2
cg18655369	chr19	1177575	
cg05242915	chr19	1263080	
cg04173586	chr19	2167496	DOT1L
cg23463608	chr19	2607757	GNG7
cg24723883	chr19	2608495	GNG7
cg26853368	chr19	2856730	
cg25919177	chr19	3355151	
cg05025071	chr19	6887530	EMR1
cg09241324	chr19	11847798	ZNF823
cg01274826	chr19	12596128	ZNF709;ZNF709
cg20078972	chr19	15391832	BRD4;BRD4
cg15845821	chr19	16830613	NWD1
cg19344626	chr19	16830749	NWD1
cg27493220	chr19	18790693	
cg21473814	chr19	18873268	CRTC1;CRTC1
cg24750752	chr19	35819889	CD22
cg05254946	chr19	39052112	RYR1;RYR1
cg09412728	chr19	40897467	HIPK4
cg11313468	chr19	41782183	HNRNPUL1;HNRNPUL1
cg19927816	chr19	46319153	SYMPK;RSPH6A
cg15229275	chr19	46800054	HIF3A
cg05286653	chr19	46800602	HIF3A;HIF3A
cg27146050	chr19	46801557	HIF3A;HIF3A
cg22891070	chr19	46801642	HIF3A;HIF3A
cg16672562	chr19	46801672	HIF3A;HIF3A;HIF3A
cg12068280	chr19	46804528	HIF3A;HIF3A
cg01552731	chr19	46806907	HIF3A;HIF3A;HIF3A;HIF3A
cg25196389	chr19	46806999	HIF3A;HIF3A;HIF3A
cg23548163	chr19	46807119	HIF3A;HIF3A;HIF3A
cg26749414	chr19	46807272	HIF3A;HIF3A;HIF3A
cg20667364	chr19	46807466	HIF3A;HIF3A;HIF3A
cg07684068	chr19	46807660	HIF3A;HIF3A;HIF3A
cg07569288	chr19	50002551	RPS11
cg16618260	chr19	50177057	BCL2L12;BCL2L12
cg16651537	chr19	51226536	CLEC11A
cg11254700	chr19	53561386	
cg19504245	chr19	55660620	TNNT1;TNNT1;TNNT1
cg18838701	chr19	55668612	TNNI3
cg27573549	chr19	56189952	EPN1;EPN1;EPN1
cg13467628	chr2	365559	
cg17494897	chr2	3200012	TSSC1
cg21547649	chr2	9142741	MBOAT2
cg23902076	chr2	9428726	ASAP2;ASAP2
cg14118850	chr2	10447890	HPCAL1;HPCAL1

cg13471188	chr2	20638399	
cg26008260	chr2	25057298	ADCY3
cg02607972	chr2	25964061	ASXL2
cg14021880	chr2	27301369	EMILIN1
cg03606774	chr2	27432830	SLC5A6;SLC5A6
cg05057534	chr2	28497669	BRE;BRE;BRE;BRE;BRE
cg04011474	chr2	28904455	
cg07671644	chr2	37545668	PRKD3
cg19190900	chr2	37553621	
cg08160072	chr2	40009501	
cg01479187	chr2	43158610	
cg10101600	chr2	43478743	THADA;THADA
cg25938803	chr2	43767347	THADA;THADA
cg02059176	chr2	44327637	
cg00782708	chr2	44933278	C2orf34
cg27485921	chr2	46747379	ATP6V1E2
cg10416784	chr2	58797233	
cg02054108	chr2	61607478	USP34
cg25521400	chr2	62445279	B3GNT2
cg26164488	chr2	64440295	
cg15742737	chr2	64863651	SERTAD2
cg22900266	chr2	65089000	
cg13814396	chr2	65090621	
cg17010895	chr2	65731517	
cg21346154	chr2	70699808	TGFA;TGFA
cg16437908	chr2	85640810	
cg05486094	chr2	85804476	VAMP8
cg14017402	chr2	86225602	
cg14057383	chr2	100636602	AFF3;AFF3
cg27315109	chr2	100677411	AFF3;AFF3
cg05991009	chr2	102080674	RFX8
cg05874806	chr2	102350276	MAP4K4;MAP4K4;MAP4K4
cg25305879	chr2	106814630	
cg24743301	chr2	112945506	FBLN7;FBLN7
cg05616969	chr2	118982661	
cg21470541	chr2	120976896	
cg06876354	chr2	121020189	RALB
cg25390440	chr2	127413369	GYPC;GYPC
cg14306819	chr2	128052889	ERCC3
cg12192282	chr2	143632190	
cg25349939	chr2	144855085	GTDC1;GTDC1;GTDC1
cg14768164	chr2	145025049	GTDC1;GTDC1
cg03167633	chr2	145338509	
cg27416489	chr2	149823115	KIF5C
cg09499849	chr2	158695158	ACVR1;ACVR1
cg08347373	chr2	160653686	CD302
cg03432176	chr2	160655066	CD302
cg01462353	chr2	169939873	DHRS9;DHRS9;DHRS9;DHRS9
cg09128944	chr2	169967580	
cg16350446	chr2	172338747	DCAF17;DCAF17;DCAF17
cg00883212	chr2	173795201	RAPGEF4;RAPGEF4
cg15058645	chr2	175528343	WIPF1
cg00405190	chr2	175545838	WIPF1
cg26466274	chr2	177864936	
cg01231381	chr2	180130081	SESTD1
cg17100158	chr2	180307728	ZNF385B;ZNF385B;ZNF385B
cg09613192	chr2	181388538	
cg17516156	chr2	191207820	INPP1;INPP1
cg04144521	chr2	191276183	MFSD6
cg26742320	chr2	201393628	SGOL2;SGOL2;SGOL2
cg02697649	chr2	202901045	FZD7
cg24206827	chr2	214098074	
cg25364972	chr2	217075573	
cg15768138	chr2	219030752	CXCR1
cg04364261	chr2	219233650	
cg19701828	chr2	219235515	
cg01827633	chr2	219610103	TTLL4
cg16786808	chr2	222434497	EPHA4

cg22175006	chr2	223757281	ACSL3;ACSL3
cg04286697	chr2	232259623	B3GNT7
cg20954977	chr2	232260116	B3GNT7
cg14012546	chr2	233981788	INPP5D;INPP5D
cg13857354	chr2	241519705	
cg13226290	chr20	1448595	NSFL1C;NSFL1C;NSFL1C
cg22042908	chr20	4802684	RASSF2
cg18952506	chr20	19916087	RIN2
cg16151538	chr20	33676388	TRPC4AP;TRPC4AP
cg24403644	chr20	42574624	TOX2;TOX2;TOX2;TOX2
cg17775490	chr20	45179354	C20orf123
cg07291836	chr20	45319455	TP53RK
cg18112953	chr20	47448545	
cg15878616	chr20	52492209	SUMO1P1
cg17398227	chr20	57582787	CTSZ
cg20278790	chr20	57583474	CTSZ
cg25957332	chr20	61562955	DIDO1;DIDO1;DIDO1
cg10615591	chr20	62318433	RTEL1;RTEL1
cg27237300	chr21	34442292	OLIG1
cg08309687	chr21	35320596	
cg15091747	chr21	36262896	RUNX1
cg17228105	chr21	39871301	ERG;ERG;ERG;ERG
cg24341911	chr21	40689521	
cg18151030	chr21	43222961	PRDM15;PRDM15
cg16624482	chr21	43548126	UMODL1;UMODL1
cg10192877	chr21	43641690	ABCG1;ABCG1;ABCG1;ABCG1;ABCG1;ABCG1
cg27243685	chr21	43642366	ABCG1;ABCG1;ABCG1;ABCG1;ABCG1;ABCG1
cg06500161	chr21	43656587	ABCG1;ABCG1;ABCG1;ABCG1;ABCG1;ABCG1
cg09354050	chr21	43824262	UBASH3A;UBASH3A
cg04217515	chr21	46325853	ITGB2;ITGB2
cg12923994	chr22	18277981	MICAL3
cg01800253	chr22	22290866	PPM1F
cg18132256	chr22	24190340	
cg26354221	chr22	24822802	ADORA2A
cg06494464	chr22	24992604	GGT1
cg01791232	chr22	29469490	KREMEN1;KREMEN1
cg06623197	chr22	30400763	MTMR3;MTMR3;MTMR3
cg01606027	chr22	31607212	LIMK2
cg08548559	chr22	31686097	PIK3IP1;PIK3IP1
cg16971128	chr22	37499859	TMPRSS6
cg03171478	chr22	37572916	
cg19360907	chr22	37977445	LGALS2
cg20686403	chr22	38438206	
cg20496314	chr22	39759864	SYNGR1;SYNGR1;SYNGR1
cg26861460	chr22	44575455	PARVG;PARVG;PARVG;PARVG
cg05249836	chr22	45609402	C22orf9;C22orf9
cg24157349	chr22	47081751	CERK
cg09349128	chr22	50327986	
cg20366239	chr22	50841775	SAPS2
cg11600734	chr3	4794020	ITPR1;ITPR1;ITPR1;EGOT
cg26269881	chr3	5023310	BHLHE40
cg00874032	chr3	5095248	
cg01564818	chr3	9464436	SETD5
cg03751527	chr3	10335375	GHRL;GHRL;GHRL;GHRL;GHRL;GHRL;GHRL;GHRL;GHRL;GHRL
cg07330114	chr3	11624023	VGLL4;VGLL4;VGLL4
cg01765641	chr3	17781888	TBC1D5;TBC1D5;TBC1D5
cg12146699	chr3	17987505	
cg09678212	chr3	41240163	CTNNB1;CTNNB1;CTNNB1
cg09417547	chr3	43289436	
cg15765353	chr3	45601455	
cg27567495	chr3	53877436	CHDH
cg01368219	chr3	54999791	CACNA2D3
cg07557173	chr3	58103186	FLNB;FLNB;FLNB;FLNB
cg22380007	chr3	59718904	
cg01556706	chr3	59804435	FHIT;FHIT
cg11377213	chr3	69370218	FRMD4B
cg15037823	chr3	71730474	EIF4E3;EIF4E3;EIF4E3;EIF4E3
cg24512093	chr3	78698151	ROBO1;ROBO1;ROBO1;ROBO1

cg10752508	chr3	98313516	CPOX
cg23528247	chr3	99832772	C3orf26;FILIP1L;FILIP1L;C3orf26
cg04244170	chr3	100116021	TOMM70A
cg12992827	chr3	101901234	
cg20672711	chr3	110965888	
cg14042799	chr3	111806031	C3orf52
cg08267038	chr3	113325595	SIDT1
cg16323245	chr3	114622118	ZBTB20;ZBTB20
cg09447811	chr3	121972621	CASR
cg14516183	chr3	122928186	SEC22A
cg18770216	chr3	124491657	ITGB5
cg08359464	chr3	128370361	RPN1
cg12405599	chr3	128370463	RPN1
cg27297137	chr3	128444572	RAB7A
cg02726291	chr3	128779596	GP9
cg00221718	chr3	128914668	
cg05936004	chr3	130693828	ATP2C1;ATP2C1;ATP2C1;ATP2C1
cg24054898	chr3	148721868	GYG1
cg21374307	chr3	159547539	SCHIP1
cg15711521	chr3	167767619	GOLIM4
cg04319611	chr3	171784422	FNDC3B;FNDC3B
cg19980260	chr3	171858472	FNDC3B;FNDC3B
cg09831562	chr3	181327125	SOX2OT
cg18030943	chr3	182876556	LAMP3
cg11341144	chr3	185656289	TRA2B
cg06390643	chr3	185775470	ETV5
cg06739107	chr3	188399479	LPP;LPP;LPP
cg25853622	chr3	188425256	LPP;LPP;LPP
cg04051365	chr3	193586394	
cg13185177	chr3	194119885	GP5
cg15431659	chr3	194901357	C3orf21
cg16834320	chr3	195167302	
cg27295342	chr3	195849368	
cg10959668	chr3	195897912	
cg03358636	chr3	197474006	KIAA0226
cg04266908	chr4	873633	GAK
cg07727358	chr4	1008738	FGFRL1;FGFRL1;FGFRL1
cg07241925	chr4	1294566	MAEA;MAEA
cg21326139	chr4	1294783	MAEA;MAEA
cg07094298	chr4	2748026	TNIP2;TNIP2
cg19224164	chr4	2966384	GRK4;NOP14;GRK4;GRK4
cg09494176	chr4	3193868	HTT
cg22358291	chr4	10101553	WDR1;WDR1
cg04332373	chr4	15779642	CD38
cg17791799	chr4	16036123	PROM1;PROM1;PROM1;PROM1;PROM1;PROM1;PROM1
cg16760587	chr4	17517594	CLRN2
cg10307548	chr4	24795830	SOD3
cg04349839	chr4	25789390	SEL1L3
cg00812557	chr4	38073835	TBC1D1
cg24279243	chr4	38676559	KLF3
cg14665413	chr4	38859728	TLR6
cg01645955	chr4	39371148	
cg08821747	chr4	39641752	C4orf34
cg20435896	chr4	40632723	RBM47
cg18463607	chr4	56718320	EXOC1;EXOC1;EXOC1
cg24746726	chr4	70726093	SULT1E1
cg14798160	chr4	74718399	PF4V1
cg04858148	chr4	81117016	PRDM8
cg03340036	chr4	89446409	PIGY;PIGY
cg04536922	chr4	89978566	FAM13A
cg15133208	chr4	90757351	SNCA;SNCA;SNCA;SNCA
cg27186013	chr4	95264127	HPGDS
cg27019278	chr4	101438804	EMCN;EMCN
cg24493367	chr4	106635507	GSTCD;GSTCD
cg22365313	chr4	108636320	PAPSS1
cg03797115	chr4	110625010	CASP6;CASP6
cg13541713	chr4	119947251	SYNPO2;SYNPO2;SYNPO2
cg23415995	chr4	128704651	HSPA4L

cg04998379	chr4	129491708	
cg03418002	chr4	140655478	MAML3
cg16218705	chr4	140941031	MAML3
cg22031873	chr4	143765657	INPP4B;INPP4B
cg03967798	chr4	145268453	
cg01400750	chr4	145956168	ANAPC10
cg06536988	chr4	153586440	TMEM154
cg17385088	chr4	153611560	
cg15410675	chr4	154144800	TRIM2;TRIM2
cg03580292	chr4	174411723	
cg21757281	chr4	183795822	
cg23576855	chr5	373299	AHRR
cg05575921	chr5	373378	AHRR
cg17287155	chr5	393347	AHRR
cg13325231	chr5	1342170	CLPTM1L
cg26333652	chr5	2750758	IRX2;IRX2
cg10530883	chr5	3596207	IRX1;IRX1
cg07712198	chr5	9015582	
cg17320136	chr5	10567905	ANKRD33B
cg08944026	chr5	10626811	ANKRD33B
cg09017434	chr5	16179660	MARCH11
cg11064966	chr5	32506514	
cg02381853	chr5	33321127	
cg26893816	chr5	33997223	AMACR;AMACR;AMACR;AMACR;AMACR
cg24893378	chr5	59837426	PART1
cg10506318	chr5	60239580	ERCC8;NDUFAF2
cg06885823	chr5	82675005	
cg01057573	chr5	118683890	TNFAIP8
cg23149687	chr5	119801643	PRR16
cg08975164	chr5	126314773	MARCH3
cg11293275	chr5	131543977	P4HA2;P4HA2;P4HA2;P4HA2;P4HA2
cg18169971	chr5	134702492	H2AFY;H2AFY;H2AFY;H2AFY
cg01874869	chr5	134735701	H2AFY;H2AFY;H2AFY;H2AFY
cg00850073	chr5	139016870	
cg27658304	chr5	139017367	
cg19628988	chr5	139040928	CXXC5
cg15165154	chr5	139041014	CXXC5
cg11176694	chr5	139050415	CXXC5
cg17656608	chr5	139486020	
cg04182865	chr5	141346431	RNF14
cg09109411	chr5	149319112	PDE6A
cg11042320	chr5	149534497	PDGFRB
cg12636435	chr5	149867965	
cg27044455	chr5	149878183	
cg00604840	chr5	154230173	C5orf4;C5orf4
cg10257049	chr5	154230308	C5orf4
cg26403843	chr5	158634085	RNF145
cg06689816	chr5	158639111	
cg18307303	chr5	158757456	IL12B;IL12B
cg26146888	chr5	159542935	PWWP2A;PWWP2A
cg12688234	chr5	169407439	FAM196B;DOCK2;FAM196B
cg00357551	chr5	169407472	FAM196B;DOCK2;FAM196B
cg25693317	chr5	171807791	SH3PXD2B
cg19072817	chr5	176057992	EIF4E1B;SNCB;SNCB;EIF4E1B
cg05614161	chr5	177627467	
cg22051146	chr5	177895047	COL23A1
cg13995774	chr5	179189810	MAML1
cg26683398	chr5	179220589	LTC4S
cg13413719	chr6	3592887	
cg03187073	chr6	4890079	CDYL;CDYL;CDYL;CDYL
cg02212836	chr6	6589075	LY86;LOC285780
cg22626169	chr6	6890951	
cg07714276	chr6	7169632	RREB1;RREB1;RREB1;RREB1
cg26514623	chr6	13295561	
cg02206980	chr6	13574034	SIRT5;SIRT5
cg03159883	chr6	14215435	
cg13746813	chr6	14911904	
cg13077031	chr6	15266782	JARID2

cg26405097	chr6	15428301	JARID2
cg24776142	chr6	16513829	ATXN1;ATXN1
cg08628635	chr6	20483859	E2F3
cg15407162	chr6	28192457	ZNF193
cg20544852	chr6	28874479	TRIM27
cg05314350	chr6	28874702	TRIM27
cg11389756	chr6	28875354	TRIM27
cg18578954	chr6	28887247	TRIM27
cg20103692	chr6	29454672	MAS1L
cg06591579	chr6	29461269	
cg10568066	chr6	30039442	RNF39;RNF39
cg08060195	chr6	30120915	TRIM10;TRIM10
cg09020199	chr6	30297320	TRIM39;TRIM39
cg04425551	chr6	30297338	TRIM39;TRIM39
cg17080697	chr6	30297382	TRIM39;TRIM39
cg01383911	chr6	30297627	TRIM39;TRIM39
cg27486585	chr6	30459595	HLA-E
cg07076751	chr6	30647539	KIAA1949;KIAA1949
cg10145196	chr6	30647649	KIAA1949;KIAA1949
cg06126421	chr6	30720080	
cg14753356	chr6	30720108	
cg19913563	chr6	30720261	
cg05631194	chr6	31146417	PSORS1C3
cg21053741	chr6	31525861	NFKBIL1;NFKBIL1;NFKBIL1;NFKBIL1
cg21587837	chr6	31525894	NFKBIL1;NFKBIL1;NFKBIL1;NFKBIL1
cg14441276	chr6	31539735	LTA;LTA
cg21999229	chr6	31540014	LTA;LTA;LTA
cg04536765	chr6	31621761	BAT3;BAT3;BAT3
cg04386400	chr6	31651158	
cg13123009	chr6	31681882	LY6G6E;LY6G6D;LY6G6E
cg25953692	chr6	31695415	DDAH2
cg16656520	chr6	31800885	
cg21587984	chr6	32295237	C6orf10
cg03639929	chr6	32765402	
cg26033526	chr6	32819858	TAP1
cg25042789	chr6	32819964	TAP1
cg17626301	chr6	32820201	TAP1
cg10123514	chr6	32904061	HLA-DMB
cg13524037	chr6	32904074	HLA-DMB
cg04903089	chr6	32905190	HLA-DMB
cg21578596	chr6	32906460	HLA-DMB
cg02015582	chr6	32918479	HLA-DMA
cg07715777	chr6	33128963	
cg04276715	chr6	33254460	WDR46;WDR46
cg26819753	chr6	33265313	RGL2;RGL2
cg12118504	chr6	33265393	RGL2;RGL2
cg14079545	chr6	33418310	SYNGAP1
cg18670721	chr6	34113570	
cg14725580	chr6	34663155	C6orf106;C6orf106
cg03546163	chr6	35654363	FKBP5;FKBP5;FKBP5;FKBP5
cg16145324	chr6	36020012	MAPK14;MAPK14;MAPK14;MAPK14
cg08110693	chr6	36407533	PXT1
cg23156989	chr6	37400664	FTSJD2
cg10852154	chr6	42421757	
cg18022921	chr6	43736973	VEGFA;VEGFA;VEGFA;VEGFA
cg03143046	chr6	43758007	
cg04609694	chr6	44204009	
cg03822267	chr6	44234126	NFKBIE
cg00171729	chr6	52416856	TRAM2
cg15807046	chr6	52438690	TRAM2
cg19701087	chr6	52859008	GSTA4
cg06311422	chr6	56406336	DST;DST;DST;DST;DST
cg00796963	chr6	84936558	KIAA1009
cg02159996	chr6	89927233	GABRR1;GABRR1
cg23746497	chr6	105388668	
cg04309234	chr6	106441468	
cg02331198	chr6	106988121	AIM1
cg01431340	chr6	110680085	C6orf186

cg26818197	chr6	110986361	CDK19
cg20222562	chr6	113993995	
cg12708994	chr6	116691460	DSE;DSE
cg16203203	chr6	125284659	STL
cg04905719	chr6	134617250	SGK1
cg27434890	chr6	135517041	MYB;MYB;MYB;MYB;MYB;MYB;MYB;MYB
cg04739200	chr6	135517046	MYB;MYB;MYB;MYB;MYB;MYB;MYB;MYB
cg24481882	chr6	136450701	PDE7B
cg00235484	chr6	136825415	MAP7
cg20865323	chr6	143999666	PHACTR2;PHACTR2;PHACTR2;PHACTR2
cg17586302	chr6	144013969	PHACTR2;PHACTR2;PHACTR2;PHACTR2
cg24690314	chr6	144018564	PHACTR2;PHACTR2;PHACTR2;PHACTR2
cg02701019	chr6	144472355	STX11
cg15785898	chr6	144608500	
cg12476487	chr6	145047472	UTRN
cg07805500	chr6	151380818	MTHFD1L
cg24900983	chr6	152128528	ESR1;ESR1;ESR1;ESR1;ESR1
cg07059469	chr6	152421432	ESR1;ESR1;ESR1;ESR1
cg16095551	chr6	155816164	
cg17164954	chr6	157345266	ARID1B;ARID1B;ARID1B
cg23206115	chr6	158066900	ZDHHC14;ZDHHC14
cg17530030	chr6	166699419	
cg17501210	chr6	166970252	RPS6KA2;RPS6KA2
cg23002590	chr6	167012092	RPS6KA2;RPS6KA2
cg16998831	chr7	187686	
cg17593625	chr7	752800	PRKAR1B;PRKAR1B;PRKAR1B;PRKAR1B;PRKAR1B;PRKAR1B;PRKAR1B
cg27572072	chr7	958244	ADAP1
cg21511321	chr7	1004997	COX19
cg04816311	chr7	1066650	C7orf50;C7orf50;C7orf50
cg17187143	chr7	1903058	MAD1L1;MAD1L1;MAD1L1
cg08972190	chr7	2138995	MAD1L1;MAD1L1;MAD1L1
cg17338816	chr7	2607424	IQCE;IQCE
cg16481332	chr7	2654053	IQCE;IQCE
cg11884933	chr7	2774414	GNA12
cg23304647	chr7	2778058	GNA12
cg16993754	chr7	3020282	CARD11
cg25344401	chr7	4755415	FOXK1
cg04336379	chr7	5111669	LOC389458
cg20367388	chr7	5258485	WIPI2;WIPI2;WIPI2;WIPI2;WIPI2
cg18237047	chr7	5276991	
cg21725496	chr7	5372402	TNRC18
cg00232092	chr7	5518887	FBXL18
cg08722383	chr7	5594654	
cg26724841	chr7	5816628	RNF216;RNF216
cg15381475	chr7	6436101	RAC1;RAC1
cg16101574	chr7	7291514	
cg04089901	chr7	7557590	COL28A1
cg11384744	chr7	16794623	TSPAN13
cg21710826	chr7	20240145	MACC1
cg17344321	chr7	22617382	
cg16954385	chr7	23246896	
cg17209188	chr7	23387396	IGF2BP3
cg02842382	chr7	27615496	HIBADH
cg13817046	chr7	29609310	
cg02181036	chr7	30328151	ZNRF2;MIR550-1
cg02060682	chr7	36190724	
cg01119452	chr7	37287850	ELMO1
cg05642546	chr7	37298927	ELMO1
cg05383910	chr7	37431792	ELMO1
cg13791719	chr7	39994617	CDK13;CDK13
cg26478599	chr7	41747322	LOC285954;LOC285954
cg07006935	chr7	55620641	VOPP1
cg21959598	chr7	55637719	VOPP1
cg01323964	chr7	65219171	SNORA22;CCT6P1
cg25444339	chr7	75194698	HIP1
cg26162326	chr7	75957061	YWHAG
cg06257058	chr7	99683264	
cg07212384	chr7	99761053	GAL3ST4

cg16914953	chr7	100798809	AP1S1
cg25711558	chr7	101499638	CUX1;CUX1;CUX1
cg02508830	chr7	102576358	FBXL13;LRRC17;FBXL13;LRRC17
cg25968394	chr7	104604511	
cg01581098	chr7	105754353	SYPL1
cg05667818	chr7	116786870	ST7;ST7;ST7OT2
cg01597480	chr7	120629404	C7orf58;C7orf58
cg17347253	chr7	130130288	MESTIT1;MEST;MEST
cg22544881	chr7	130712346	FLJ43663;FLJ43663
cg18463686	chr7	141646690	CLEC5A;CLEC5A
cg01844514	chr7	149557121	ZNF862
cg00323915	chr7	150264987	GIMAP4
cg03450844	chr7	150416671	GIMAP1
cg21887193	chr7	150786082	AGAP3;AGAP3
cg09449988	chr7	151328116	PRKAG2;PRKAG2;PRKAG2
cg04598292	chr7	158495856	NCAPG2
cg27048067	chr8	674560	ERICH1
cg01607321	chr8	1872335	ARHGEF10
cg09147140	chr8	1924394	KBTBD11
cg20165746	chr8	8870185	ERI1
cg01535726	chr8	9911482	MSRA;MSRA
cg17560136	chr8	21915510	EPB49;EPB49;EPB49;EPB49;EPB49;EPB49
cg24805258	chr8	22423091	SORBS3;SORBS3
cg23221723	chr8	22963367	TNFRSF10C
cg09069499	chr8	26216910	PPP2R2A
cg10789956	chr8	26467541	DPYSL2
cg01008602	chr8	27144085	TRIM35
cg22313574	chr8	27468981	CLU;CLU;CLU
cg14917244	chr8	27469001	CLU;CLU;CLU
cg03746834	chr8	28196926	PNOC
cg25596405	chr8	28961333	KIF13B
cg20106866	chr8	29082964	KIF13B
cg11638399	chr8	29441416	
cg05965490	chr8	30264627	RBPMS;RBPMS;RBPMS;RBPMS
cg04589674	chr8	38831508	HTRA4
cg04771100	chr8	38832335	HTRA4
cg16230352	chr8	42123158	
cg27320734	chr8	48744559	PRKDC;PRKDC
cg02098075	chr8	49643972	EFCAB1;EFCAB1;EFCAB1
cg21301224	chr8	56813677	LYN;LYN
cg17347634	chr8	65711916	CYP7B1
cg07896832	chr8	67975874	CSPP1;CSPP1;COPS5
cg21320567	chr8	67975880	CSPP1;CSPP1;COPS5
cg03706086	chr8	71381216	
cg12112870	chr8	86112931	E2F5;E2F5;E2F5
cg18507018	chr8	92035517	TMEM55A
cg07152894	chr8	102373664	
cg24023489	chr8	103129227	NCALD;NCALD;NCALD;NCALD;NCALD;NCALD;NCALD
cg20611272	chr8	103548145	
cg10011091	chr8	104424477	SLC25A32
cg17679987	chr8	106535882	ZFPM2
cg08165796	chr8	117484298	
cg05783185	chr8	119121281	EXT1
cg26196626	chr8	121989053	
cg21597811	chr8	123687197	
cg03314365	chr8	123798823	ZHX2
cg26140475	chr8	126525558	
cg14752089	chr8	128773042	
cg24514600	chr8	128805414	PVT1
cg11201447	chr8	128808063	MIR1204;PVT1
cg20612299	chr8	131308633	ASAP1IT1;ASAP1
cg20513418	chr8	131457460	
cg10726725	chr8	140930736	TRAPPC9;TRAPPC9
cg09859659	chr8	142180109	DENND3
cg17207736	chr8	142237307	SLC45A4
cg17159187	chr8	144420118	
cg13469851	chr9	26936447	PLAA
cg14363249	chr9	75213807	TMC1

cg13438337	chr9	78774369	PCSK5
cg14113203	chr9	97346942	FBP2
cg13717434	chr9	97854044	
cg13753351	chr9	127134207	PSMB7
cg13982956	chr9	128521976	PBX3;PBX3;PBX3;PBX3
cg21234265	chr9	132370351	
cg14611767	chr9	134127575	
cg01944288	chr9	135036217	NTNG2
