# Supplementary material for: An epigenome-wide association study in whole blood of measures of adiposity among Ghanaians: the RODAM study
Source: Clin Epigenetics. 2017 Sep 21;9:103. doi: 10.1186/s13148-017-0403-x (PMC5609006; doi:10.1186/s13148-017-0403-x)
Supplement: Additional file 1: — Additional methods, quality control, sensitivity analyses and supplementary results. (ZIP 1243 kb) [file 13148_2017_403_MOESM1_ESM.zip › 13148_2017_403_MOESM1_ESM/Supplementary Files_revised - CLEP-D-17-00109.pdf]

Additional files

File S1. Quality control figures and tables

Table S1. R packages used and their versions

R version:	3.3.1
R studio version:	0.99.896
<u>Packages:</u>	
limma:	3.28.5
ChAMP:	1.10.0
minfi:	1.18.2
bumphunter:	1.12.0
FlowSorted.Blood.450k:	1.10.0
Illumina450ProbeVariants.db:	1.8.0
IlluminaHumanMethylation450kanno.ilmn12.hg19:	0.2.1
IlluminaHumanMethylation450kmanifest:	0.4.0
methylaid:	1.6.2
<u>Other/extra:</u>	
affy:	1.50.0
Biobase:	2.32.0
BioGenerics:	0.18.0
Biostrings:	2.40.1
ChAMPdata:	1.10.0
doParallel:	1.0.10
foreach:	1.4.3
GenomeInfoDb:	1.8.1
GenomicRanges:	1.24.0
Iranges:	2.6.0
iterators:	1.0.8
locfit:	1.5-9.1
minfiData:	0.14.0
S4Vectors:	0.10.1
SummarizedExperiment:	1.2.2
Xvector:	0.12.0
lattice:	0.20-33

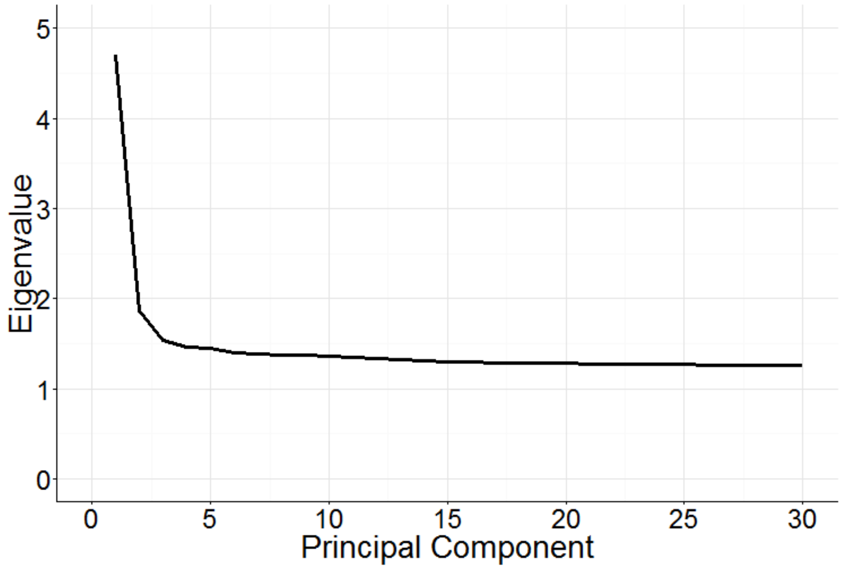

Figure S1-A. Scree plot of eigenvalues for the first 30 principal components of genotyping analysis.

The % explained variance for the first 3 PCs was 0.44%

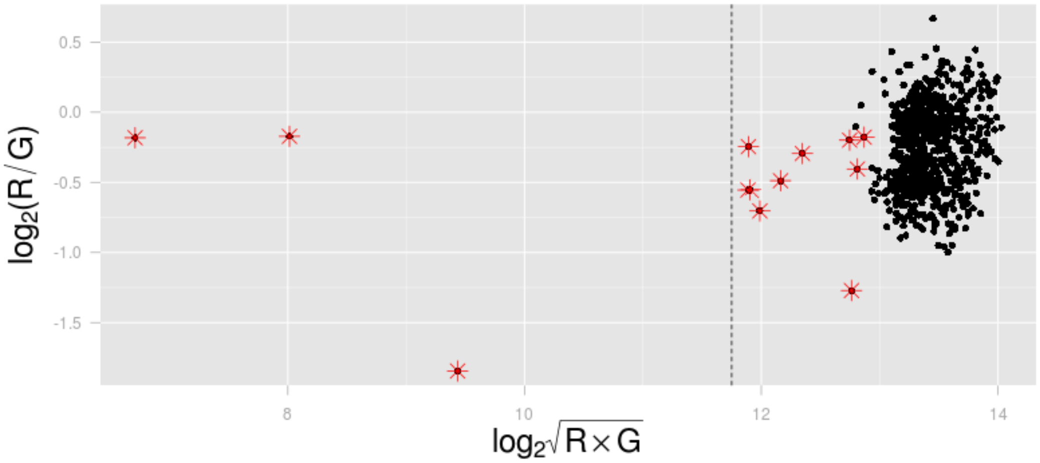

Figure S1-B. Sample dependent overall quality control in *MethylAid*. Red samples were flagged as outliers and were excluded.

R = Red signal, G = Green signal

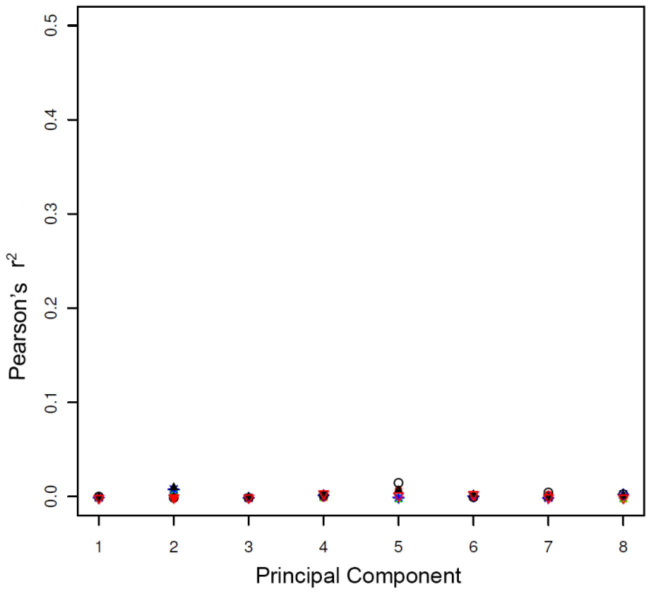

Figure S1-C. Correlation blood cell count covariates with principal component 1 to 8.

Green square = CD8+ T cells, Red circle = CD4+ T cells, Blue asterisk = Natural Killer cells, white circle = B cells, black triangle = Granulocytes, Red triangle = Monocytes

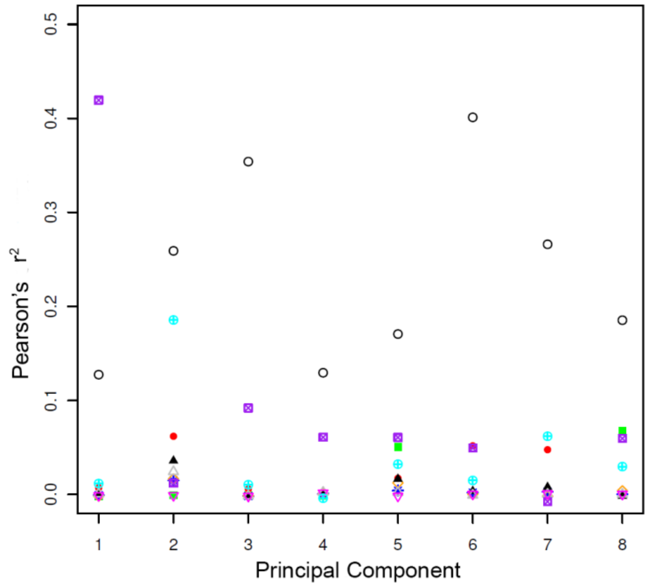

Figure S1-D. Correlation covariates with principal component 1 to 8.

Green square = sex, red circle = age, blue asterisk = obesity, orange diamond = abdominal obesity, white circle = hybridization batch, black triangle = body mass index, grey triangle = plate position, purple square = waist circumference, pink triangle = genotyping PC1, light blue circle = site.

File S2. Evaluation of model fitting

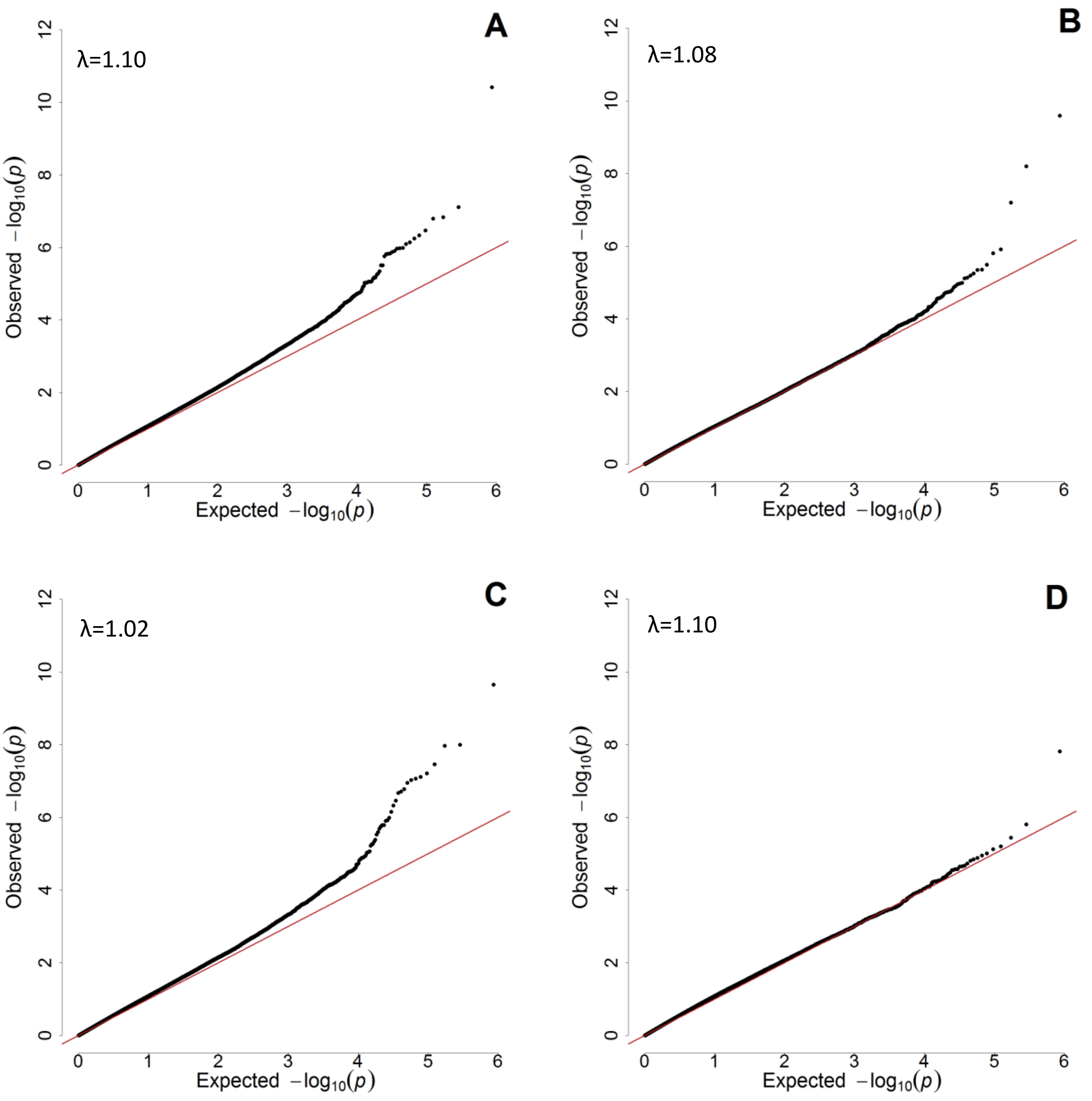

Figure S2. QQ plots of EWAS p-values for (a) BMI, (b) obesity, (c) waist circumference and (d) abdominal obesity.

File S3. Evaluation of methods to reduce inflation in EWAS

To explore methods to minimize residual inflation of our results we implemented and applied the "R" package *BACON* (version 1.4.0) entitled "Controlling bias and inflation in association studies using the empirical null distribution" using default settings. This method was applied to our dataset in order to perform a inflation correction that was recently described by Iterson *et al* (2017)[1].

Evaluating the both sets of QQ plots (Supplementary Figure S3), we conclude that the shape of the QQ plot does not substantially improve. Furthermore, evaluating Supplementary Table S3-A and Table S3-B we observe that the DMPs identified in the manuscript remain significant after BACON implementation. In addition, several FDR adjusted P-values become slightly more significant.

QQ plots *prior* (as in Supplementary File S2)	QQ plots *post* method to correct for inflation
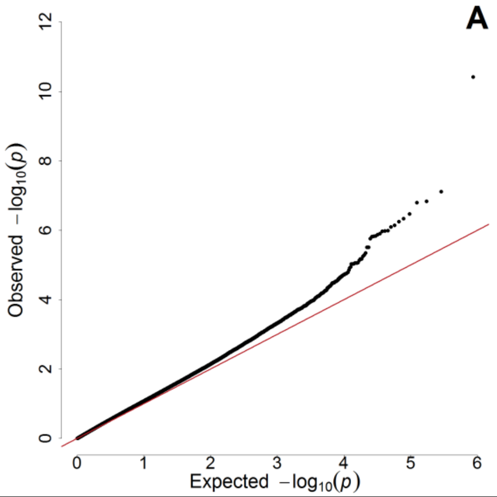	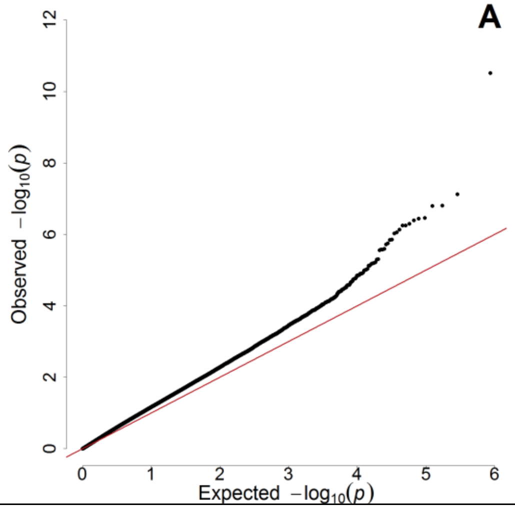
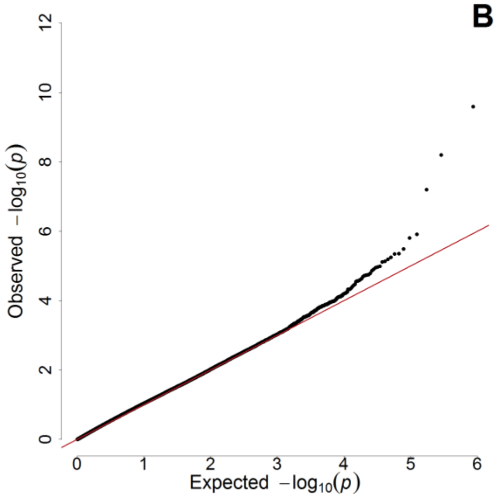	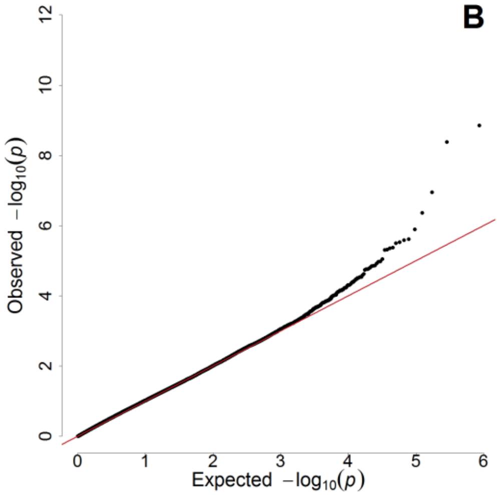

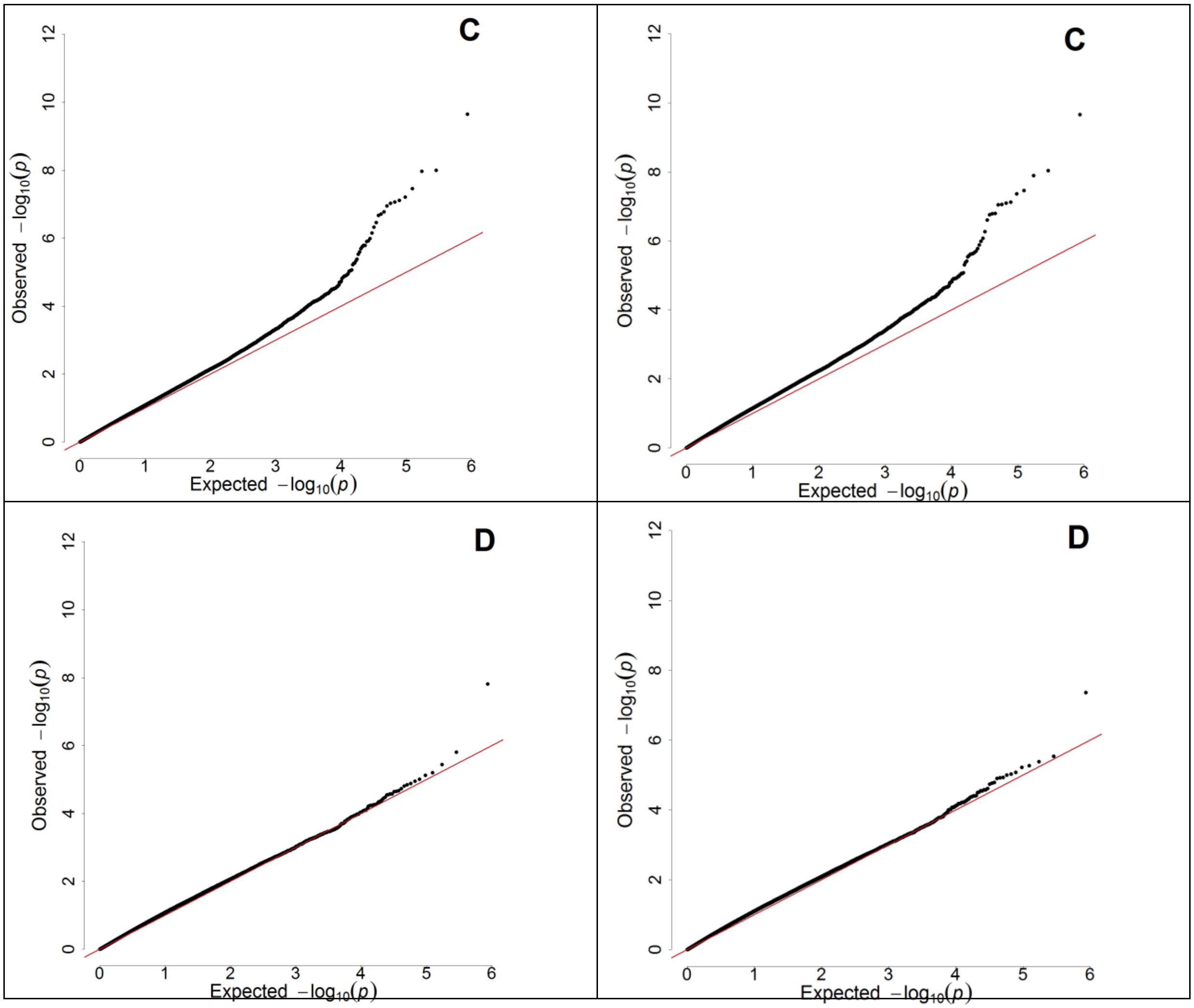

Figure S3. QQ plots of EWAS p-values prior (left column) and post (right column) implementation of methods to reduce inflation in EWAS.
A = BMI, B = obesity, C = waist circumference, D = abdominal obesity

Table S3-A. Comparison of results for BMI and obesity prior (original) and post (BACON) implementation of method to reduce inflation in EWAS

CpG site	CHR	Position	Nearest gene[a]	Feature[b]	BMI		Obesity	
					FDR original[c]	FDR BACON[c]	FDR original[c]	FDR BACON[c]
cg07839457	16	57023022	NLRC5	TSS1500	1.63E-05	8.20E-06	1.37E-03	1.58E-04
cg08818207	6	32820355	TAP1	Body	1.64E-02	4.68E-03	2.67E-01	6.21E-02
cg00574958	11	68607622	CPT1A	5'UTR	1.71E-02	3.92E-05	1.09E-04	1.10E-07
cg08099136	6	32811251	PSMB8	Body	1.71E-02	8.25E-03	6.62E-01	2.11E-01
cg01309328	6	32811253	PSMB8	Body	2.91E-02	1.15E-02	3.19E-01	6.87E-02
cg20399616	12	25055967	BCAT1	Body	3.30E-02	9.43E-05	9.10E-03	2.41E-05
cg22107533	15	45028083	TRIM69	TSS1500	3.49E-02	1.39E-02	6.41E-01	2.24E-01
cg06820412	5	135386296	*TGFBI*	Body	3.81E-02	2.50E-02	3.71E-01	2.24E-01
cg00218406	6	31431407	*HCP5*	3'UTR	3.81E-02	1.56E-02	5.33E-01	1.60E-01
cg25954539	6	31323677	*HLA-B*	Body	3.81E-02	1.64E-02	4.15E-01	1.24E-01
cg23235965	6	30459540	*HLA-E*	Body	3.81E-02	2.01E-02	2.42E-01	6.15E-02
cg25178683	17	76976267	*LGALS3BP*	TSS1500	3.81E-02	1.39E-02	6.41E-01	2.09E-01
cg08996521	3	50649994	*CISH*	TSS1500	3.81E-02	2.01E-02	3.19E-01	6.21E-02
cg04927537	17	76976091	*LGALS3BP*	TSS200	3.81E-02	1.71E-02	2.76E-01	6.87E-02
cg05490029	8	79719015	*IL7*	TSS1500	3.81E-02	1.39E-02	3.71E-01	9.58E-02
cg18954700	10	124220854	*HTRA1*	TSS200	3.81E-02	5.31E-04	8.19E-01	1.60E-01
cg25843003	6	31431312	*HCP5*	3'UTR	3.81E-02	2.25E-02	6.22E-01	1.66E-01
cg06118217	2	240100998	*HDAC4*	Body	4.11E-02	1.17E-02	3.19E-01	1.09E-01

a. *CpG's are located in the gene if no distance is indicated.*
b. *Based on manifest feature annotation Illumina. IGR = Intergenic region.*
c. *FDR corresponding to M-values. Table is sorted on BMI associated p-values for the original method. All significant hits for both outcomes are included.*

Table S3-B. Comparison of results for waist circumference and abdominal obesity prior (original) and post (BACON) implementation of method to reduce inflation in EWAS

ACpG site	CHR	Position	Nearest gene[a]	Feature[b]	WC		Abdominal obesity	
					FDR original[c]	FDR BACON[c]	FDR original[c]	FDR BACON[c]
cg07839457	16	57023022	*NLRC5*	TSS1500	9.61E-05	1.94E-05	8.17E-01	9.20E-01
cg00574958	11	68607622	*CPT1A*	5'UTR	1.55E-03	9.35E-07	6.54E-03	3.51E-04
cg25954539	6	31323677	*HLA-B*	Body	1.55E-03	5.37E-04	7.75E-01	9.20E-01
cg08818207	6	32820355	*TAP1*	Body	3.71E-03	1.35E-03	7.75E-01	8.90E-01
cg04927537	17	76976091	*LGALS3BP*	TSS200	5.08E-03	1.58E-03	7.75E-01	8.90E-01
cg22107533	15	45028083	*TRIM69*	TSS1500	5.08E-03	2.13E-03	8.17E-01	9.60E-01
cg01309328	6	32811253	*PSMB8*	Body	5.08E-03	2.13E-03	6.45E-01	8.42E-01
cg23533285	6	31322348	*HLA-B*	Body	5.08E-03	2.13E-03	8.17E-01	9.60E-01
cg23570810	11	315102	*IFITM1*	Body	5.35E-03	2.13E-03	6.68E-01	8.42E-01
cg00218406	6	31431407	*HCP5*	3'UTR	7.12E-03	3.53E-03	8.17E-01	9.60E-01
cg08099136	6	32811251	*PSMB8*	Body	7.53E-03	3.53E-03	8.17E-01	9.60E-01
cg11202345	17	76976057	*LGALS3BP*	1stExon	7.70E-03	4.75E-03	7.75E-01	8.90E-01
cg25178683	17	76976267	*LGALS3BP*	TSS1500	1.16E-02	3.53E-03	6.68E-01	8.42E-01
cg01971407	11	313624	*IFITM1*	TSS1500	1.44E-02	7.61E-03	7.75E-01	9.20E-01
cg25843003	6	31431312	*HCP5*	3'UTR	2.01E-02	1.04E-02	8.17E-01	9.20E-01
cg22940798	6	32805554	*TAP2*	Body	2.75E-02	1.75E-02	8.17E-01	9.60E-01
cg20399616	12	25055967	*BCAT1*	Body	2.97E-02	2.07E-04	8.17E-01	9.20E-01
cg05439368	15	45028098	*TRIM69*	TSS1500	2.97E-02	1.16E-02	8.17E-01	9.60E-01
cg06538684	12	12511223	*LOH12CR2*	TSS1500	3.53E-02	1.50E-02	8.17E-01	9.43E-01
cg23235965	6	30459540	*HLA-E*	Body	3.53E-02	1.74E-02	7.75E-01	9.20E-01
cg13558971	1	203597085	*ATP2B4*	5'UTR	3.64E-02	2.87E-04	8.17E-01	9.60E-01
cg13348877	18	78005237	*PARD6G*	1stExon	3.93E-02	2.83E-04	8.17E-01	8.42E-01
cg08996521	3	50649994	*CISH*	TSS1500	4.70E-02	2.01E-02	8.17E-01	9.20E-01

a. *CpG's are located in the gene if no distance is indicated.*
b. *Based on manifest feature annotation Illumina. IGR = Intergenic region.*
c. *FDR corresponding to M-values. Table is sorted on waist circumference associated p-values for the original method. All significant hits for both outcomes are included.*

References File S3

1. van Iterson M, van Zwet EW, Heijmans BT. Controlling bias and inflation in epigenome-and transcriptome-wide association studies using the empirical null distribution. Genome biology. 2017;18:1.

File S4. Search strategy in PUBMED and flow chart for inclusion of articles

(obesity[MeSH Terms] OR abdominal obesity[MeSH Terms] OR body mass index[MeSH Terms] OR obesity[Title/Abstract] OR body mass index[Title/Abstract] OR abdominal obesity[Title/Abstract] OR waist[Title/Abstract] OR waist to hip ratio[MeSH Terms] OR waist to hip ratio[Title/Abstract]) AND (epigenetics[MeSH Terms] OR dna methylation[MeSH Terms] OR epigenetics[Title/Abstract] OR methylation[Title/Abstract] OR 450k[Title/Abstract] OR 27k[Title/Abstract]) AND ("2013/01/01"[Date - Publication] : "3000"[Date - Publication]) NOT (mice[Title/Abstract] OR murine[Title/Abstract] OR rodent[Title/Abstract] OR zebrafish[Title/Abstract] OR rat[Title/Abstract] OR bariatric surgery[MeSH Terms] OR surgery[Title/Abstract] OR children [Title/Abstract])

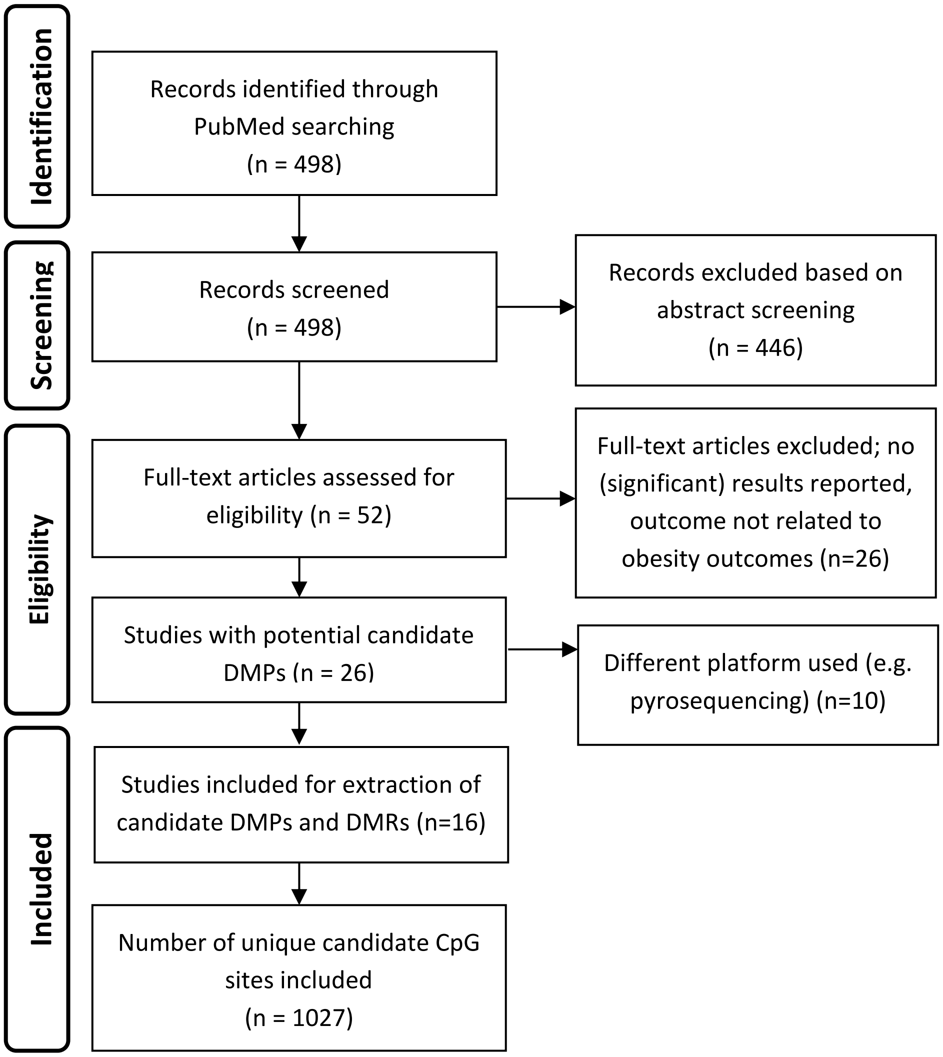

Figure S4. PRISMA flow chart for inclusion of articles

File S5. List of unique probes for adiposity identified through systematic literature search
(Separate PDF)

This PDF holds a list of previously reported 450k CpG sites associated with adiposity (n=1027) derived using a systematic literature search on published literature in PUBMED. The search was performed in April 2016. The first column in the supplemental PDF is the probe name, the second and third column are corresponding chromosome and position respectively. Column 4 is the UCSC reference gene name (hg 19).

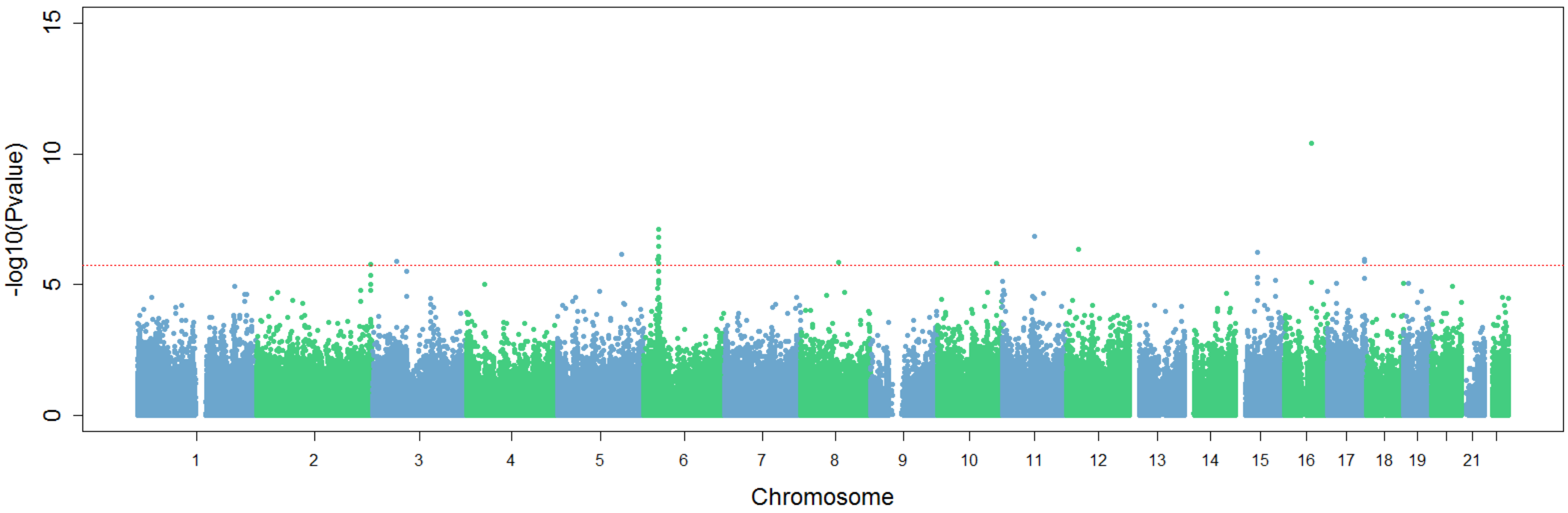

Figure S6. Manhattan plot of epigenome wide p-values for BMI

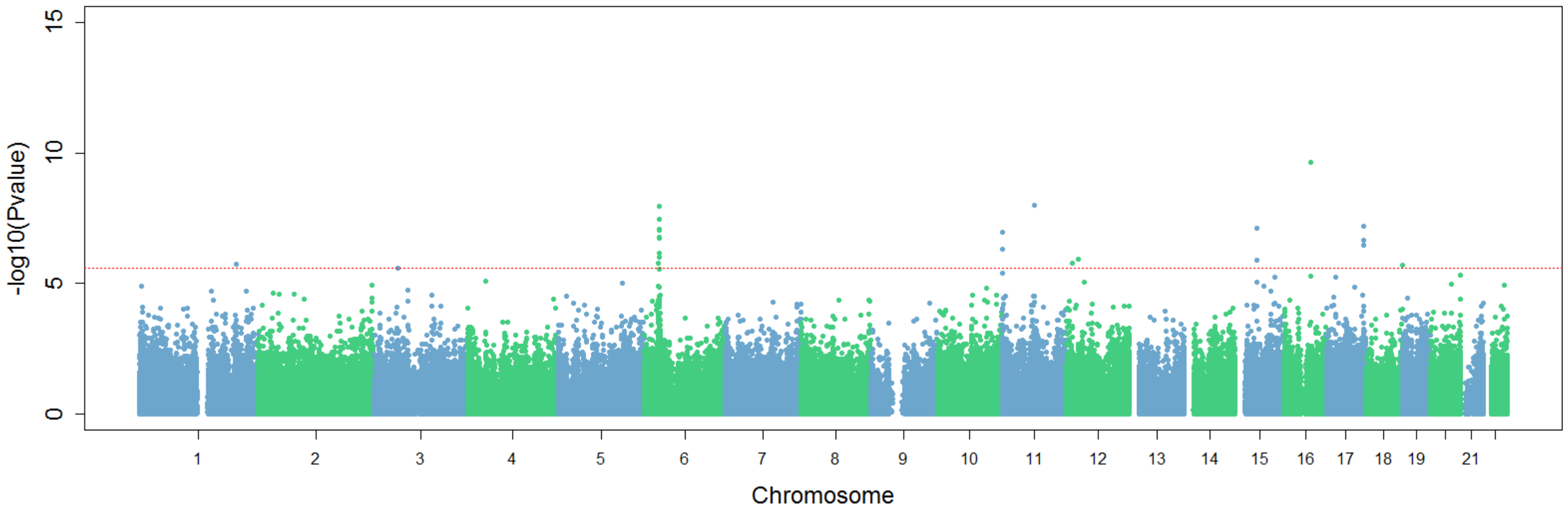

Figure S7. Manhattan plot of epigenome wide p-values for WC

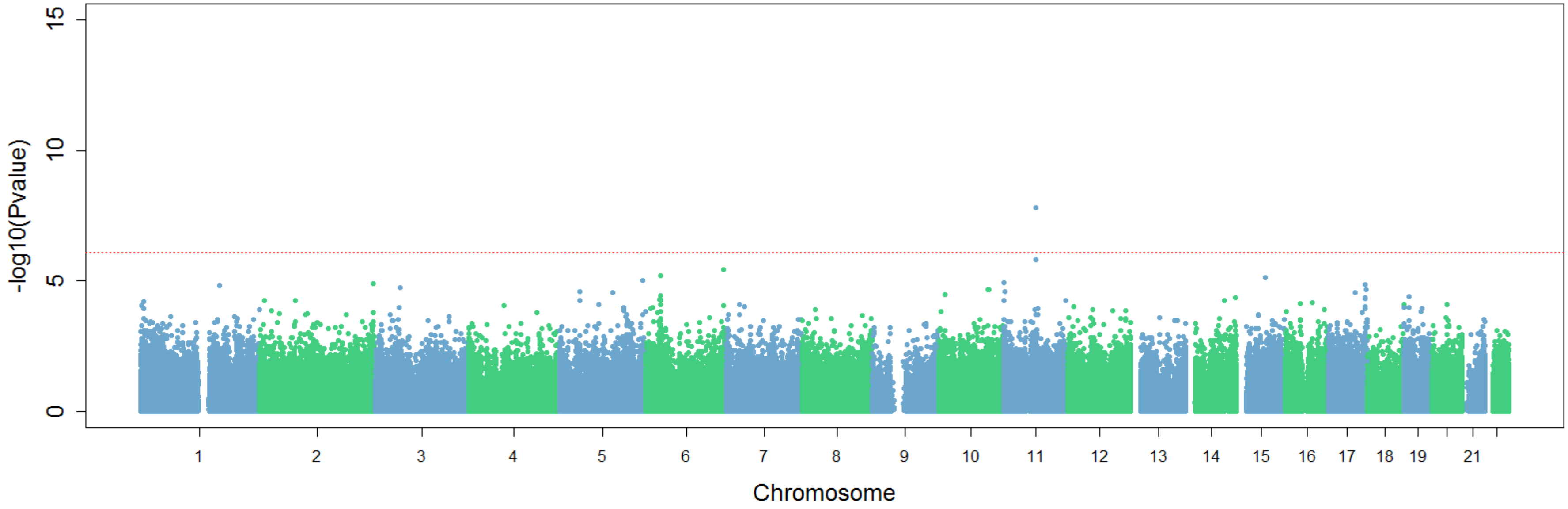

Figure S8. Manhattan plot of epigenome wide p-values for abdominal obesity (WC ≥88cm women or ≥102cm men)
The red dotted line indicates epigenome-wide significance according to FDR multiple test correction.

File S9. Obesity and abdominal obesity Differentially Methylated Positions (DMPs) adjusted energy intake and physical activity

Energy intake was assessed by means of a standardised Food Propensity Questionnaire developed specifically for Ghanaian populations, based on the European Food Propensity Questionnaire[1]. It queried the usual frequency of food intake during the past 12 months. The WHO STEPS questionnaire[2] was used to derive physical activity in metabolic equivalent (MET) h/week, which included physical activity at work, while commuting and in leisure time.

Table S9. Odds ratios for Obesity and Abdominal Obesity DMPs adjusted and unadjusted for energy intake and physical activity

					**Model 1[a]**			**Model 2[b]**		
CpG	CHR	Position	Gene[c]	Feature[d]	OR[e]	95%CI	Attributable trait variance	OR[e]	95%CI	Attributable trait variance
**Obesity**										
cg07839457	16	57023022	*NLRC5*	TSS1500	1.04	1.02-1.06	2.4%	1.04	1.01-1.06	2.4%
cg00574958	11	68607622	*CPT1A*	5'UTR	0.85	0.80-0.91	6.1%	0.85	0.79-0.90	7.0%
cg20399616	12	25055967	*BCAT1*	Body	0.94	0.90-0.98	1.6%	0.94	0.89-0.98	2.1%
**Abdominal obesity**										
cg07839457	16	57023022	*NLRC5*	TSS1500	1.03	1.01-1.06	1.4%	1.04	1.02-1.07	2.5%
cg00574958	11	68607622	*CPT1A*	5'UTR	0.84	0.79-0.90	5.6%	0.83	0.77-0.90	6.6%
cg20399616	12	25055967	*BCAT1*	Body	0.96	0.91-1.00	0.7%	0.95	0.90-1.00	1.1%

a. *Model 1 = Adjusted for age + sex + site of data collection + estimated cell distributions + technical effects*
b. *Model 2 = Model 1 + energy intake (kcal) + physical activity (MET h/week)*
c. *CpG's are located in the gene if no distance is indicated.*
d. *Based on manifest feature annotation Illumina.*
e. Odds ratios are per 1% increase in DNA methylation.

References File S9

1. Kaaks R, Riboli E (1997) Validation and calibration of dietary intake measurements in the EPIC project: methodological considerations. European Prospective Investigation into Cancer and Nutrition. International Journal of Epidemiology 26: S15-S25
2. World Health Organization (2005) WHO STEPS surveillance manual: the WHO STEPwise approach to chronic disease risk factor surveillance. Available from www.who.int/chp/steps/en/, accessed 14 April 2016.

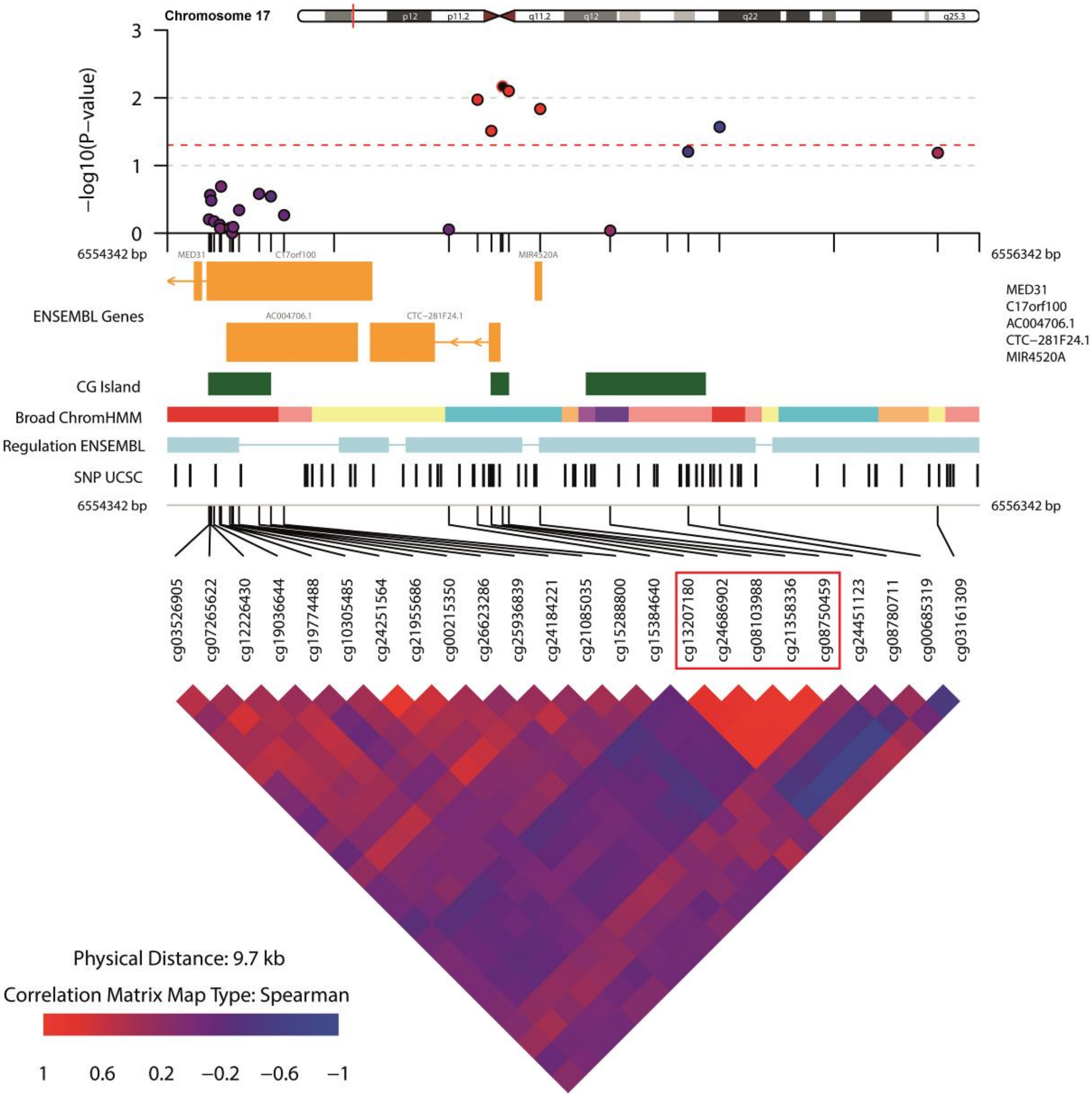

Figure S10. Differentially Methylated Region (DMR) annotated to chromosome 17 (*MIR4520A*) associated with obesity

The DMR consists of the CpG sites in the red square. A 5kb region around the DMR was visualized.

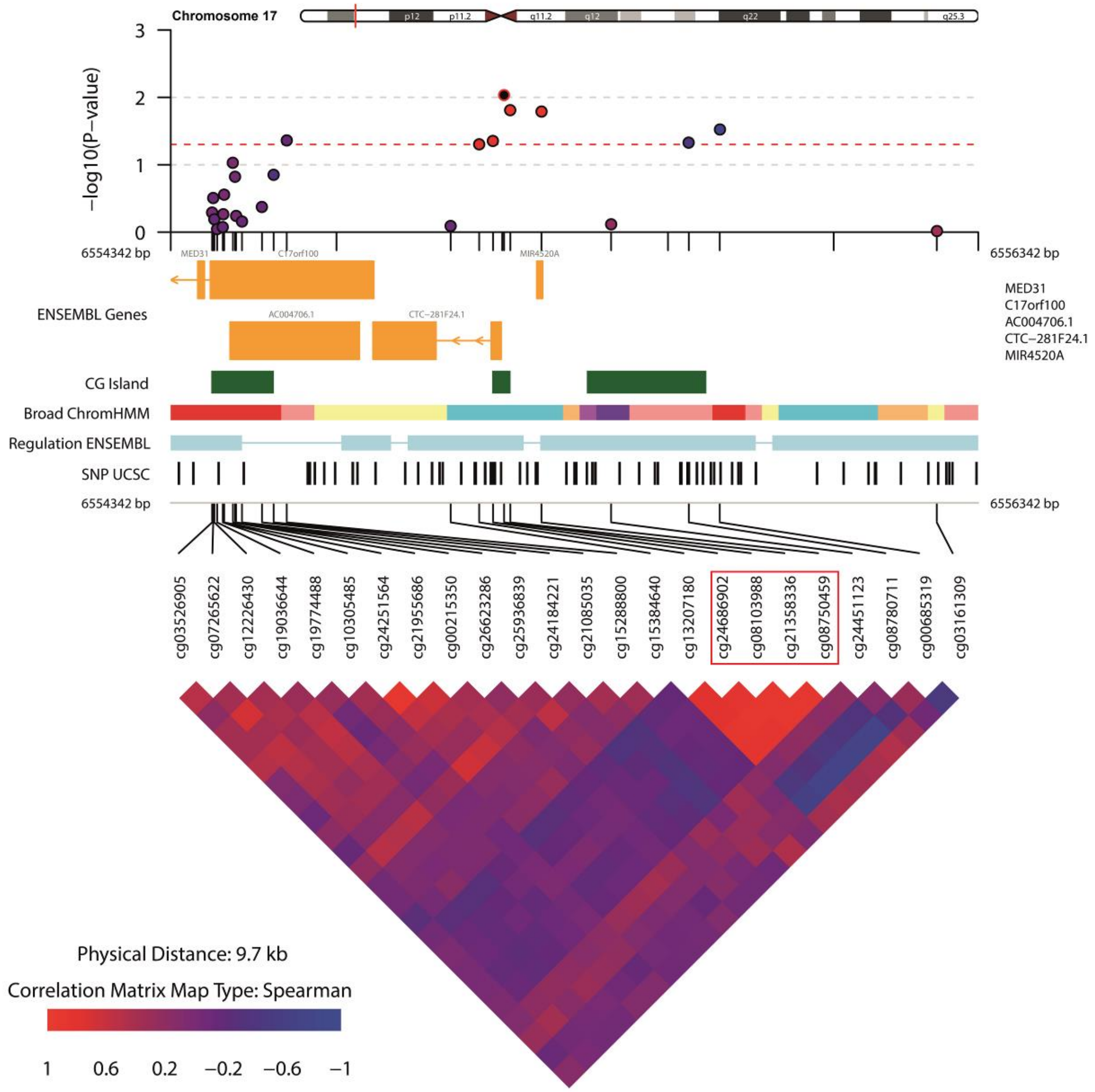

Figure S11. Differentially Methylated Region (DMR) annotated to chromosome 17 (*MIR4520A*) associated with abdominal obesity

The DMR consists of the CpG sites in the red square. A 5kb region around the DMR was visualized.

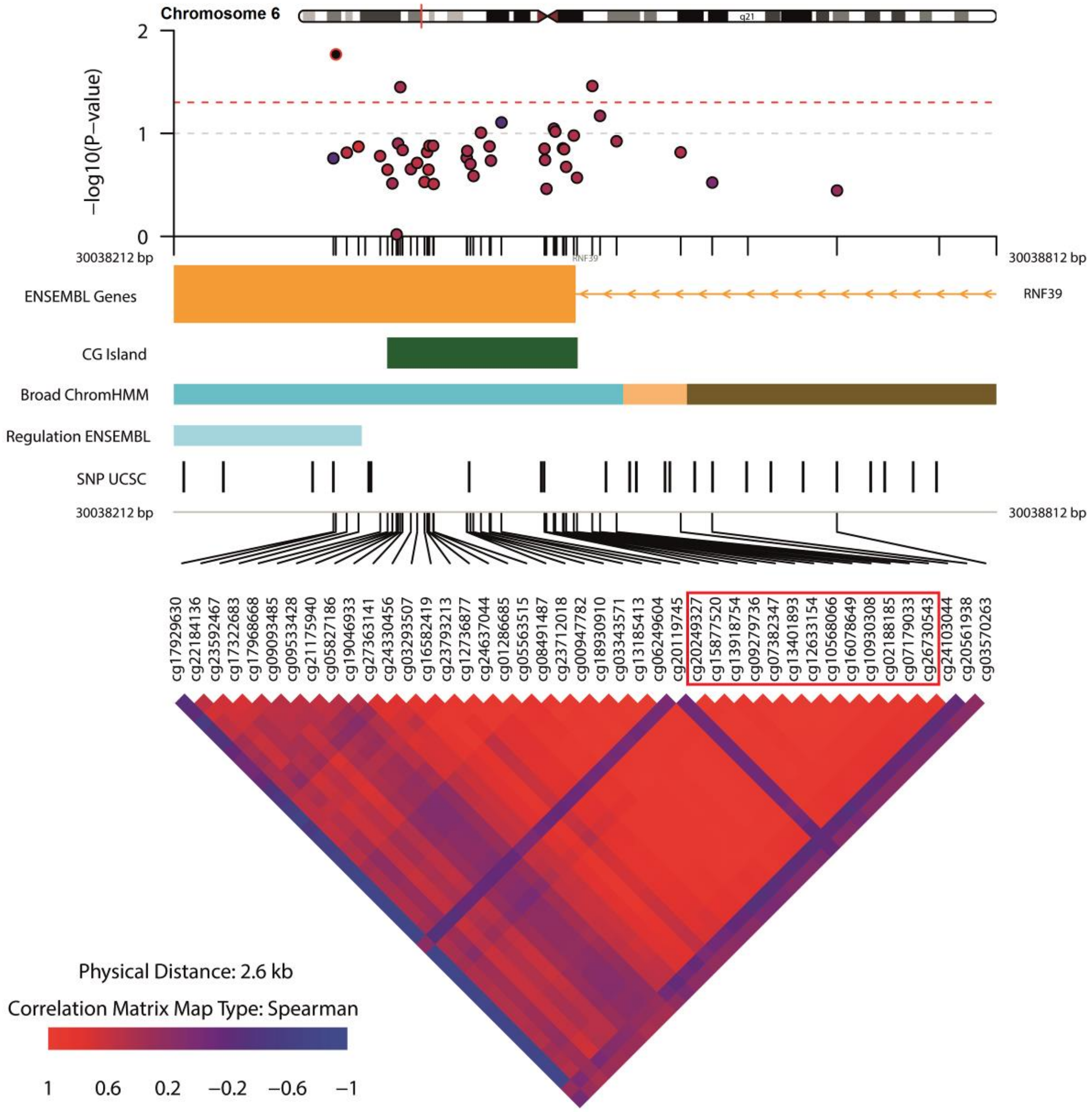

Figure S12. Differentially Methylated Region (DMR) annotated to chromosome 6 (*RNF39*) associated with obesity

The DMR consists of the CpG sites in the red square. A 1kb region around the DMR was visualized.

File S13. Pathway analysis

Table S13. Pathways including Differentially Methylated Positions (DMPs) associated with BMI

Pathway name	Pathway ID	Database	Genes in pathway	C	O	E	R	p-value	FDR
response to type I interferon	0034340	BP	HLA-B, PSMB8, HLA-E, IFITM1, **NLRC5**	73	5	0.30	16.70	1.19E-05	4.00E-04
cellular response to type I interferon	0071357	BP	HLA-B, PSMB8, HLA-E, IFITM1, **NLRC5**	72	5	0.30	16.93	1.11E-05	4.00E-04
type I interferon-mediated signaling pathway	0060337	BP	HLA-B, PSMB8, HLA-E, IFITM1, **NLRC5**	72	5	0.30	16	1.11E-05	4.00E-04
regulation of response to stimulus	0048583	BP	ARAP3, SEMA4C, HTRA1, HLA-E, GPR65, TAP1, SIX3, ITGA5, GATA3, FYB, IFITM1, RHOU, HLA-B, CISH, BIRC7, RPH3AL, CNIH3, **NLRC5**, TAP2, HMGA1, PROK2, ATP6V1H, PARP9	2336	23	9.58	2.40	2.37E-05	7.00E-03
response to interferon-gamma	0034341	BP	HLA-B, HLA-E, IFITM1, **NLRC5**, PARP9	109	5	0.45	11.19	8.25E-05	1.40E-03
immune system process	0002376	BP	IL7, NCF4, HLA-E, GPR65, TAP1, ITGA5, GATA3, RORA, FYB, IFITM1, HLA-B, PSMB8, HDAC4, **NLRC5**, TAP2, ATP6V1H, CTSD, CXCR1, PARP9	1792	19	7.35	2.59	6.05E-05	1.40E-03
defense response	0006952	BP	HLA-B, PSMB8, HCP5, HDAC4, HLA-E, **NLRC5**, TAP1, GATA3, PROK2, ATP6V1H, CXCR1, IFITM1, LGALS3BP, PARP9	1107	14	4.54	3.08	1.00E-04	1.40E-03
regulation of immune system process	0002682	BP	IL7, HLA-B, HLA-E, **NLRC5**, TAP1, GATA3, TAP2, ATP6V1H, RORA, FYB, IFITM1, PARP9	865	12	3.55	3.38	2.00E-04	2.60E-03
response to cytokine stimulus	0034097	BP	HLA-B, PSMB8, HDAC4, HLA-E, **NLRC5**, GATA3, CXCR1, IFITM1, PARP9	506	9	2.08	4.34	2.00E-04	2.60E-03
cell communication	0007154	BP	ARAP3, SEMA4C, HTRA1, SH3GL3, HLA-E, GPR65, GATA3, RBPJL, RORA, LGALS3BP, IFITM1, NAMPT, CISH, PSMB8, BIRC7,	4770	33	19.5 6	1.69	3.00E-04	3.60E-03

			RPH3AL, CNIH3, SREBF1, ATP6V1H, IL7, NCF4, ITGA5, SIX3, KCNQ1, **CPT1A**, FYB, RHOU, HLA-B, HDAC4, **NLRC5**, PROK2, SH3PXD2A, CXCR1						
immune response	0006955	BP	IL7, HLA-B, NCF4, PSMB8, HLA-E, PSMB8, HLA-E, GPR65, **NLRC5**, TAP1, GATA3, TAP2, FYB, IFITM1, PARP9	1071	13	4.39	2.96	3.00E-04	3.60E-03
regulation of immune response	0050776	BP	HLA-B, HLA-E, **NLRC5**, TAP1, GATA3, TAP2, IFITM1, FYB, PARP9	555	9	2.28	3.95	4.00E-04	4.60E-03
response to stimulus	0050896	BP	ARAP3, SEMA4C, HTRA1, SH3GL3, HLA-E, GPR65, TAP1, RBPJL, GATA3, RORA, LGALS3BP, IFITM1, NAMPT, CISH, PSMB8, RPH3AL, BIRC7, CNIH3, SREBF1, TAP2, ATP6V1H, PARP9, IL7, NCF4, ITGA5, SIX3, KCNQ1, TGFBI, **CPT1A**, FYB, RHOU, HLA-B, HCP5, HDAC4, OR51I1, **NLRC5**, HMGA1, PROK2, CXCR1, ATPB4	6636	40	27.2 2	1.47	7.00E-04	7.60E-01
cell surface receptor signaling pathway	0007166	BP	SEMA4C, HTRA1, HLA-E, GPR65, SIX3, ITGA5, GATA3, FYB, IFITM1, NAMPT, HLA-B, CISH, PSMB8, RPH3AL, CNIH3, **NLRC5**, SREBF1, PROK2, ATP6V1H, CXCR1	2325	20	9.54	2.10	7.00E-04	7.50E-03
single-organism process	0044699	BP	ARAP3, PCOLCE, BIRC6, SEMA4C, HTRA1, SH3GL3, HLA-E, GPR65, VENTX, TAP1, RBPJL, GATA3, RORA, LGALS3BP, IFITM1, NAMPT, CISH, PSMB8, RPH3AL, BIRC7, CNIH3, SREBF1, TAP2, CTSD, ATP6V1H, IL7, NCF4, ITGA5, SIX3, KCNQ1, TGFBI, **CPT1A**, FYB, ROBO4, RHOU, HLA-B, SLC26A7, HDAC4, OR51I1, **NLRC5**, PROK2, CXCR1, PURA, ATP2B4	7682	44	31.5 1	1.40	8.00E-04	8.50E-03
signaling	0023052	BP	IL7, ARAP3, SEMA4C, HTRA1, SH3GL3, HLA-E, GPR65, SIX3, ITGA5, GATA3, RBPJL, KCNQ1, **CPT1A**, RORA, FYB,	4646	31	19.0 5	1.63	1.00E-03	1.00E-02

			LGALS3BP, IFITM1, NAMPT, RHOU, HLA-B, CISH, PSMB8, HDAC4, BIRC7, RPH3AL, CNIH3, **NLRC5**, SREBF1, PROK2, ATP6V1H, CXCR1						
single organism signaling	0044700	BP	IL7, ARAP3, SEMA4C, HTRA1, SH3GL3, HLA-E, GPR65, SIX3, ITGA5, GATA3, RBPJL, KCNQ1, **CPT1A**, RORA, FYB, LGALS3BP, IFITM1, NAMPT, RHOU, HLA-B, CISH, PSMB8, HDAC4, BIRC7, RPH3AL, CNIH3, **NLRC5**, SREBF1, PROK2, ATP6V1H, CXCR1	4646	31	19.05	1.63	1.00E-03	1.00E-02
peptide transport	0015833	BP	TAP2, KCNQ1, **CPT1A**, SREBF1, TAP1	199	5	0.82	6.13	1.30E-03	1.27E-02
cellular response to cytokine stimulus	0071345	BP	GATA3, HLA-B, PSMB8, CXCR1, HLA-E, IFITM1, **NLRC5**	411	7	1.69	4.15	1.40E-03	1.33E-02
sequence-specific DNA binding	0043565	MF	HDAC4, VENTX, **NLRC5**, SREBF1, GATA3, RBPJL, SIX3, HMGA1, PURA, RORA	680	10	2.62	3.81	2.00E-04	8.00E-03
transcription regulatory region DNA binding	0044212	MF	RBPJL, GATA3, HDAC4, PURA, **NLRC5**, SREBF1	328	6	1.27	4.74	1.60E-03	1.38E-02
regulatory region DNA binding	0000975	MF	RBPJL, GATA3, HDAC4, PURA, **NLRC5**, SREBF1	336	6	1.30	4.63	1.80E-03	1.38E-02
regulatory region nucleic acid binding	0001067	MF	RBPJL, GATA3, HDAC4, PURA, **NLRC5**, SREBF1	336	6	1.30	4.63	1.80E-03	1.38E-02
integral to organelle membrane	0031301	CC	HLA-B, TAP2, **CPT1A**, HLA-E, TAP1	208	5	0.81	6.21	1.30E-03	1.35E-02
intrinsic to organelle membrane	0031300	CC	HLA-B, TAP2, **CPT1A**, HLA-E, TAP1	230	5	0.89	5.62	2.00E-03	1.84E-02
cytosol	0005829	CC	ARAP3, NCF4, PARD6G, HTRA1, TAP1, VARS, **BCAT1**, FYB, NAMPT, RHOU, CISH, PSMB8, HDAC4, **NLRC5**, HMGA1, ATP6V1H, GIMAP4, PARP9	2372	18	9.18	1.96	3.20E-03	2.41E-02

Genome-wide significant DMPs for obesity are highlighted in bold. BP: Biological Process, CC: Cellular Component and MF: Molecular Function. C:

the number of reference genes in the category, O: the number of genes in the gene set and also in the category, E: the expected number in the category, R: ratio of enrichment, p-value: p value from hypergeometric test, FDR: p value adjusted by the FDR multiple test adjustment.

Table S13-B. Pathways including Differentially Methylated Positions (DMPs) associated with WC

Pathway name	Pathway ID	Database	Genes in pathway	C	O	E	R	p-value	FDR
positive regulation of biological process	0048518	BP	IL7, GAL, LMO1, BIRC6, SEMA4C, TAP1, SIX3, ITGA5, SEMA7A, RBPJL, PSMB9, **CPT1A**, RORA, IFITM1, DE3A, IER3, PSMB8, HDAC4, BIRC7, FGFR2, BASP1, NLRC5, TAP2, HMGA1, PROK2, GPIHBP1	3439	26	13.87	1.87	4.00E-04	4.00E-03
peptide transport	0015833	BP	GAL, TAP2, KCNQ1, **CPT1A**, TAP1	199	5	0.80	6.23	1.20E-03	1.05E-02
amide transport	0042886	BP	GAL, TAP2, KCNQ1, **CPT1A**, TAP1	207	5	0.83	5.99	1.50E-03	1.25E-02
intrinsic to organelle membrane	0031300	CC	HLA-B, TAP2, HLA-C, **CPT1A**, HLA-E, TAP1	230	6	0.85	7.06	2.00E-04	2.60E-03
integral to organelle membrane	0031301	CC	HLA-B, TAP2, HLA-C, **CPT1A**, HLA-E, TAP1	208	6	0.77	7.81	1.00E-04	2.60E-03

Genome-wide significant DMPs for abdominal obesity are highlighted in bold. BP: Biological Process, CC: Cellular Component and MF: Molecular Function. C: the number of reference genes in the category, O: the number of genes in the gene set and also in the category, E: the expected number in the category, R: ratio of enrichment, p-value: p value from hypergeometric test, FDR: p value adjusted by the FDR multiple test adjustment.
